# EpCAM^+^ Extracellular Vesicle PD‐L1 Dynamics as a Predictive Biomarker of Immune Checkpoint Blockade Response

**DOI:** 10.1002/advs.76389

**Published:** 2026-07-03

**Authors:** Byeonggeol Mun, Jinyoung Kim, Chang Gon Kim, Seokhyeong Go, Mina Han, Yujin Ouck, Soojin Jang, Seong Uk Son, Gamin Kim, Wonrak Son, Eunjung Kim, Min Hee Hong, Ja Hyun Yeo, Eun‐Kyung Lim, Hye Ryun Kim, Seungjoo Haam

**Affiliations:** ^1^ Department of Chemical and Biomolecular Engineering College of Engineering Yonsei University Seoul Republic of Korea; ^2^ Bionanotechnology Research Center Korea Research Institute of Bioscience and Biotechnology (KRIBB) Daejeon Republic of Korea; ^3^ Division of Medical Oncology Department of Internal Medicine Yonsei University College of Medicine Seoul Republic of Korea; ^4^ Department of Nanobiotechnology KRIBB School of Biotechnology University of Science and Technology Daejeon Republic of Korea; ^5^ Department of Bioengineering and Nano‐Bioengineering Research Center for Bio Materials and Process Development Incheon National University Incheon Republic of Korea; ^6^ School of Pharmacy Sungkyunkwan University Suwon Republic of Korea; ^7^ YUHS‐KRIBB Medical Convergence Research Institute Yonsei University Seoul Republic of Korea; ^8^ Graduate School of Medical Science Brain Korea 21 Project Yonsei University College of Medicine Seoul Republic of Korea

**Keywords:** EpCAM‐positive extracellular vesicles, head and neck squamous cell carcinomas, immune checkpoint blockades, liquid biopsies, non‐small‐cell lung cancers, programmed death‐ligand 1, surface‐enhanced Raman scattering

## Abstract

Programmed death‐ligand 1 (PD‐L1) blockade improves outcomes in patients with various malignancies; however, biomarkers for monitoring treatment responses are lacking. This study presents a surface‐enhanced Raman scattering (SERS)‐based platform for the ultrasensitive detection of PD‐L1 on circulating epithelial cell adhesion molecule‐positive (EpCAM^+^) extracellular vesicles (EVs). The platform is validated using interferon‐gamma‐treated epithelial tumor cell line‐derived EVs. The platform is further applied to paired pre‐ and post‐treatment plasma samples from patients with non‐small‐cell lung cancer (*n* = 140) and head and neck squamous cell carcinoma (*n* = 73) receiving anti‐PD‐(L)1 immunotherapy. Dynamic changes in PD‐L1 expression on EV are shown to correlate with clinical outcomes. A post‐treatment decrease in PD‐L1 expression on EpCAM^+^ EVs (EpCAM^+^ EV PD‐L1) is associated with improved 5‐year progression‐free and overall survival. These results establish EpCAM^+^ EV PD‐L1 as a dynamic, non‐invasive biomarker for monitoring immunotherapy responses and demonstrate the utility of SERS‐based profiling in predicting long‐term responses to immune checkpoint blockade.

## Graphical abstract

A highly sensitive SERS‐based platform measures PD‐L1 expression on EpCAM^+^ circulating EVs from cancer patients undergoing immunotherapy. Monitoring dynamic changes after treatment reveals that reduced EpCAM^+^ EV PD‐L1 levels associate with improved long‐term clinical outcomes, establishing a non‐invasive approach for tracking immune checkpoint inhibitor responses through liquid biopsy.

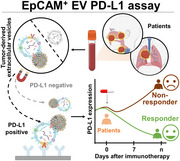

## 1 Introduction

Programmed death‐ligand 1 (PD‐L1) blockade is applicable to immunotherapy for several solid tumors, such as melanoma, renal cell carcinoma, gastric cancer, and breast cancer, as well as specific hematologic malignancies like classical Hodgkin lymphoma, and is a particularly appropriate treatment for non‐small cell lung cancer (NSCLC) and head and neck squamous cell carcinoma (HNSCC) [1, 2, 3, 4, 5]. Although they differ in anatomical origin, these tumors share key immunobiological features, including frequent PD‐L1 overexpression and a reliance on immune evasion for tumor progression [6, 7, 8]. Immune checkpoint inhibitors targeting PD‐(L)1 have demonstrated substantial clinical benefit in subsets of NSCLC and HNSCC patients; however, responses remain variable and unpredictable [9, 10, 11, 12]. Identifying robust biomarkers of treatment efficacy remain a challenge in the management of these diseases. Epithelial cell adhesion molecule (EpCAM) has gained attention as a marker of epithelial tumor cells, closely associated with cancer stemness, tumor progression, and immune modulation [13, 14, 15, 16, 17, 18]. Its high and specific expression in various tumors renders it a valuable target for monitoring tumor‐derived signals in circulation and evaluating dynamic behavior during immunotherapy [19, 20, 21].

Recent studies have focused on tumor‐derived extracellular vesicles (EVs) as carriers of molecular information about the tumor microenvironment [17, 22, 23, 24, 25, 26, 27]. Tumor‐ and immune cell‐derived EV‐associated PD‐L1 modulates systemic immune responses and has been proposed as a surrogate marker of therapeutic response [19, 28, 29, 30, 31, 32, 33, 34]. Because of their relative stability in peripheral circulation, EVs offer an attractive, minimally invasive window into real‐time tumor–immune dynamics [24, 35, 36, 37, 38]. However, the clinical utility of EV‐based biomarkers is constrained by the limitations of existing detection platforms [36, 39, 40, 41, 42]. Conventional protein detection methods, such as enzyme‐linked immunosorbent assay and western blotting, often lack the sensitivity and specificity required to quantify low‐abundance antigens [43, 44, 45, 46]. These limitations hinder real‐time monitoring of immune checkpoint dynamics and delay the integration of EV‐based markers into clinical decision‐making. Thus, advanced analytical platforms with the sensitivity and specificity required to detect EV‐associated PD‐L1 expression and associated immune signatures are urgently needed. Such platforms would enable dynamic monitoring of immunotherapy response across cancer types, especially in NSCLC and HNSCC, for which repeated tissue biopsies are impractical.

Surface‐enhanced Raman scattering (SERS) has emerged as a powerful technique for ultrasensitive molecular detection [44, 47, 48, 49]. Leveraging the plasmonic enhancement of Raman signals by engineered metallic nanostructures, SERS enables the detection of targets at femtomolar to attomolar concentrations, far exceeding the detection limits of conventional optical methods [45, 50, 51]. When integrated with immunoaffinity capture, SERS can identify and quantify specific EV‐associated proteins, such as PD‐L1, with high accuracy, even in complex biological fluids, such as plasma [28, 52, 53].

**FIGURE 1:**
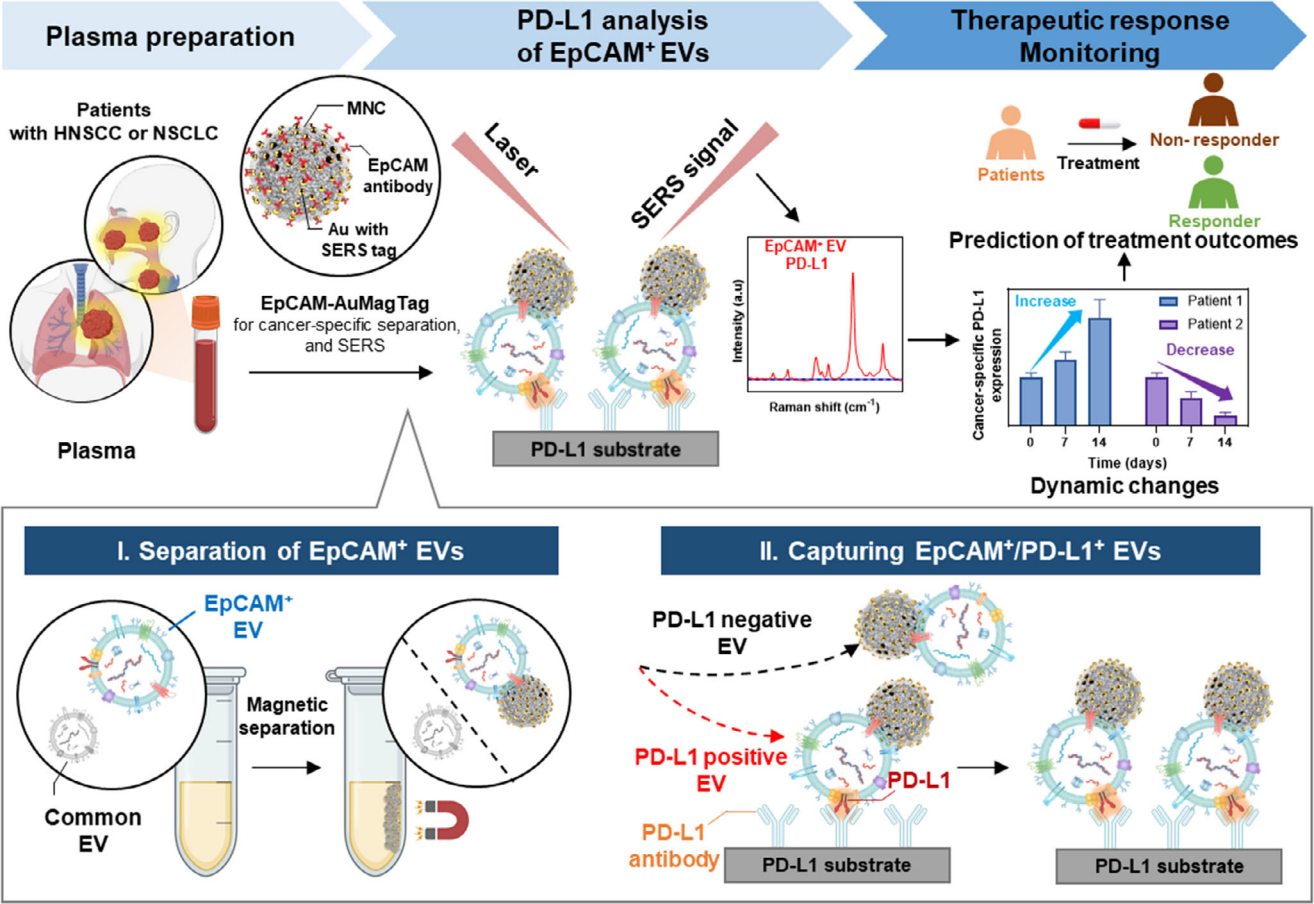
Schematic of the EpCAM^+^ extracellular vesicle (EV) PD‐L1 assay. Circulating EpCAM^+^ EVs were isolated from plasma using antibody‐conjugated gold‐coated magnetic nanoparticles tagged with a Raman reporter (EpCAM–AuMagTags) and transferred to an antibody‐functionalized substrate. PD‐L1 expression was measured through SERS spectroscopy. This platform enables the dynamic monitoring of immunotherapy response in patients with NSCLC and HNSCC through minimally invasive sampling.

In the present study, we developed a SERS platform for ultrasensitive detection of PD‐L1 expression on circulating EpCAM‐positive (EpCAM^+^) EVs by integrating magnetic immunocapture with plasmonic amplification (Figure 1). For proof of concept and validation, interferon‐gamma (IFN‐γ)‐treated epithelial tumor cell line (A549, PC‐9, FaDu, and SCC152)‐derived EVs, in which PD‐L1 expression was artificially induced, were used. The SERS platform was applied to patient‐derived plasma samples to monitor dynamic changes in PD‐L1 expression on EpCAM^+^ EVs during PD‐(L)1 immunotherapy. This approach was designed to explore the clinical relevance of non‐invasive EV‐based immune monitoring of multiple epithelial cancers.

## 2 Results

### 2.1 Nanoparticle and Substrate Preparation

**FIGURE 2:**
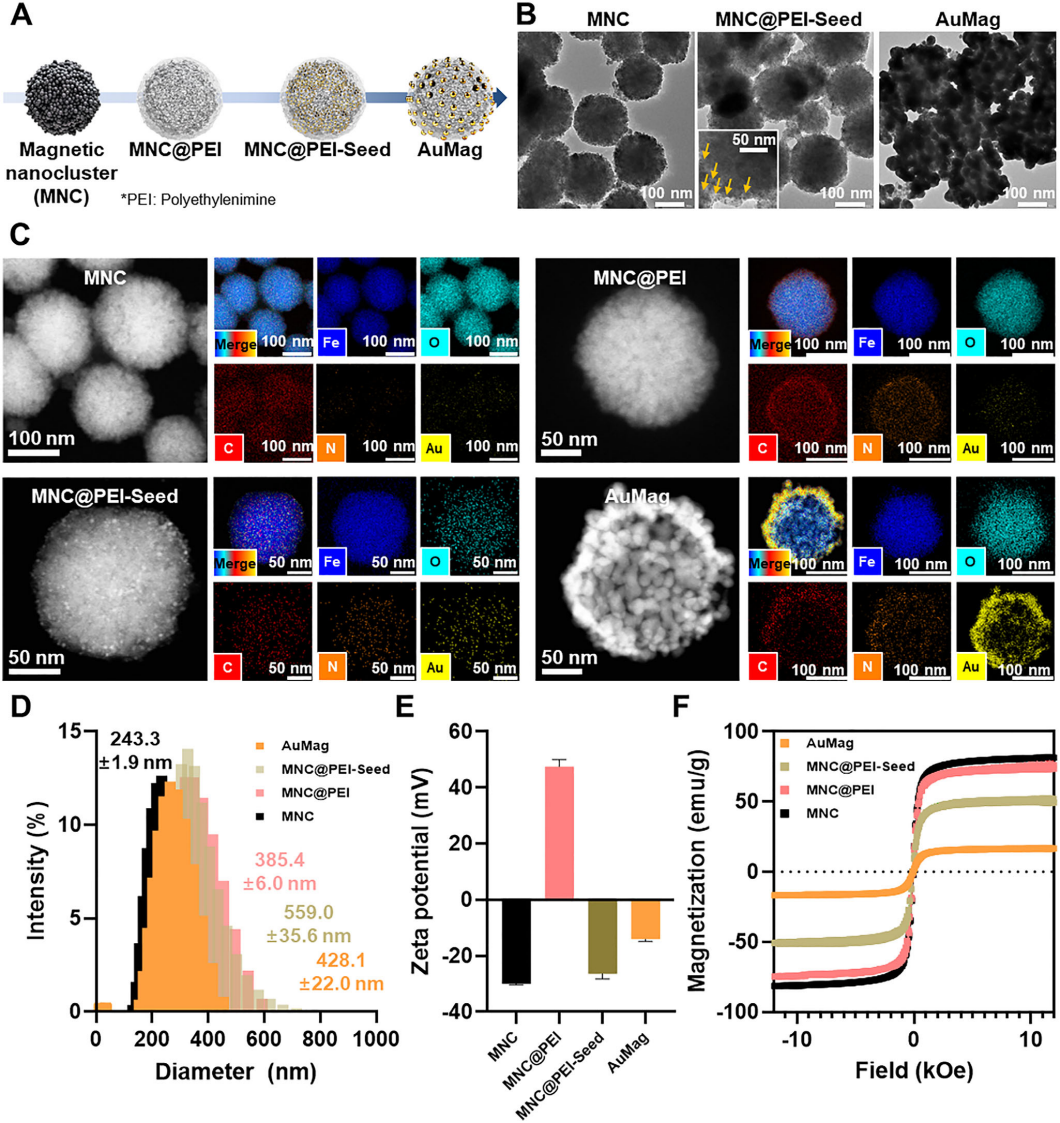
Characterization of AuMags. (A) Schematic of the synthesis of AuMags using the seed‐mediated growth method. (B) TEM and (C) EDS mapping of bare, PEI‐coated, Au‐seed‐functionalized, and Au‐coated MNCs. (D) Hydrodynamic size distributions. (E) Zeta potential at each stage of the synthesis. (F) Magnetic hysteresis loops comparing magnetic properties throughout the coating process.

Plasmonic gold‐coated magnetic nanoparticles (AuMags) designed to capture and separate EpCAM^+^ extracellular vesicles (EVs) from plasma were synthesized (Figure 2a). Magnetic nanoclusters (MNCs) were synthesized via a solvothermal reaction [54, 55, 56]. To facilitate subsequent surface modification, the MNCs were coated with polyethylenimine (PEI), forming positively charged PEI‐coated MNCs (MNC@PEIs). Gold seeds (2 nm gold nanoparticles) were electrostatically attached to the surface of the MNC@PEIs (seed‐conjugated MNC@PEIs [MNC@PEI‐Seeds]), which acts as a stable core for subsequent gold growth. AuMags were obtained by reducing gold ions in the presence of polyvinylpyrrolidone as a surfactant and ascorbic acid as a reducing agent. The morphology of the synthesized nanoparticles was examined using transmission electron microscopy (TEM) (Figure 2b). Gold seeds were visible on the MNC@PEI surface. Subsequent gold growth resulted in the formation of gold domains. Energy‐dispersive X‐ray spectroscopy (EDS) confirmed the structure of the AuMags, consisting of a Fe_3_O_4_ magnetic core with surface‐localized gold nanoparticles (Figure 2c). The spatial segregation of Au and Fe signals in EDS mapping confirmed the formation of the AuMags, crucial for decoupling magnetic enrichment and SERS readout functionalities. The hydrodynamic size of the nanoparticles (MNCs: 243.3 ± 1.9 nm; MNC@PEIs: 385.4 ± 6.0 nm; MNC@PEI‐Seeds: 559.0 ± 35.6 nm; AuMags: 428.1 ± 22.0 nm) was determined using dynamic light scattering (Figure 2d). The corresponding change in zeta potential—from negative (MNC: −30.0 ± 0.3 mV) to strongly positive (MNC@PEIs: 47.5 ± 2.5 mV) and then moderately negative (MNC@PEI‐Seeds: −26.4 ± 1.8 mV; AuMags: ‐14.0 ± 0.8 mV)—indicated successful surface modification (Figure 2e). The magnetic hysteresis loops showed that the AuMags retained their superparamagnetic properties despite the antiferromagnetic properties of the gold surface (Figure 2f). With a saturation magnetization of 17 emu g^−1^, the AuMags demonstrated sufficient magnetic responsiveness for EV separation. The X‐ray diffraction patterns showed peaks consistent with both Fe_3_O_4_ (JCPDS No. 75‐1609) and crystalline gold (JCPDS No. 04‐0784), confirming the successful synthesis of AuMags (Figure S1a). The presence of Au 111 and 200 peaks and the absence of Fe_3_O_4_‐related Bragg reflections indicated the successful coating of the magnetic core with a gold shell. The UV–vis spectrum of AuMags exhibited a distinct localized surface plasmon resonance (LSPR) peak at 550 nm, demonstrating their suitability for SERS excitation (Figure S1b). The AuMags were colloidally stable in various media, including phosphate‐buffered saline (PBS), cell culture medium, serum, and red blood cell solution, without visible aggregation. The AuMags maintained stability even after magnetic separation and redispersion, highlighting their potential for physiological applications (Figure S1c).

**FIGURE 3:**
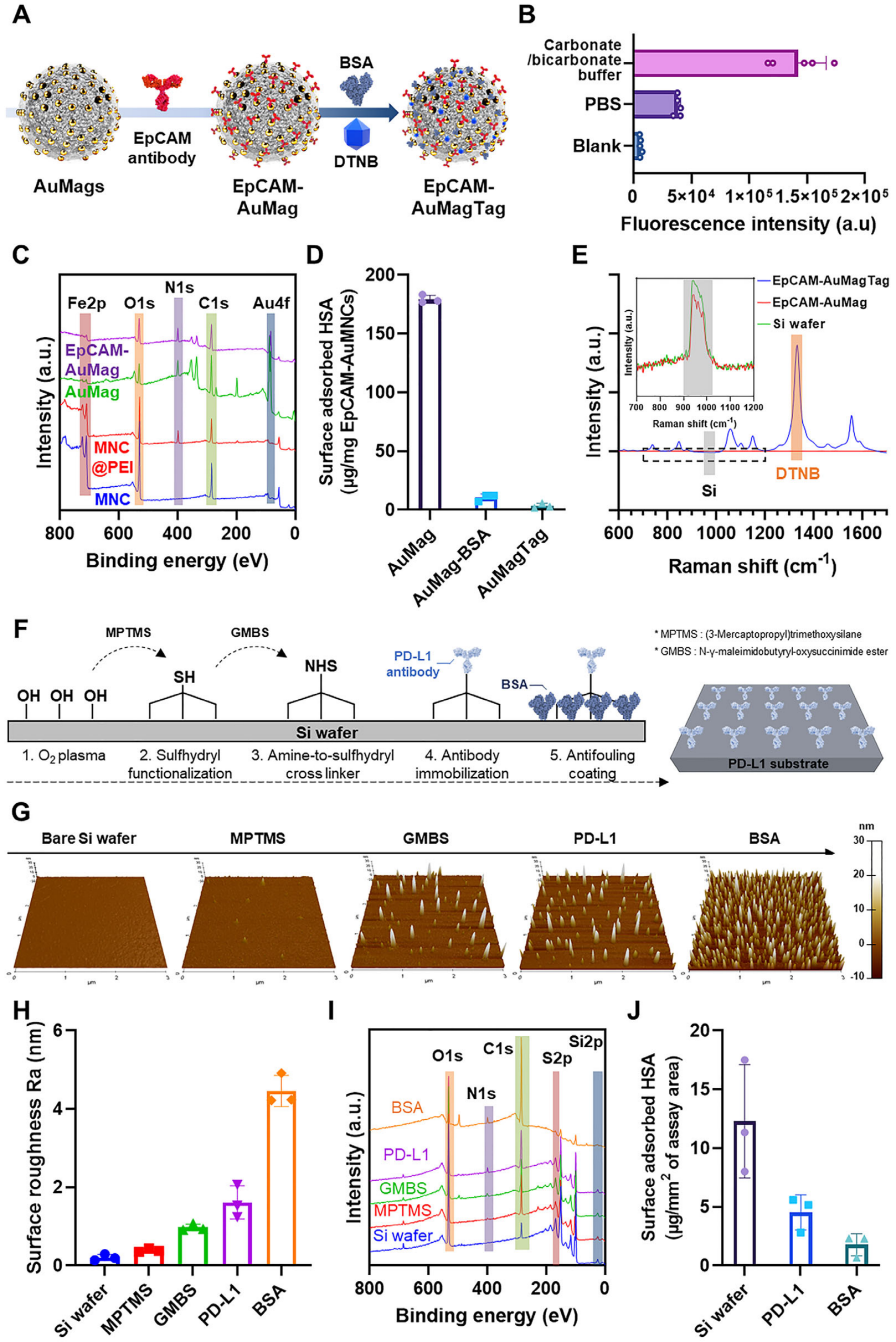
Preparation of EpCAM–AuMagTag and PD‐L1 substrate for SERS‐based detection of PD‐L1 on extracellular vesicles. (A) Schematic of the synthesis of EpCAM–AuMagTags. (B) Comparison of the fluorescence intensity of the PE‐conjugated antibody in carbonate/bicarbonate buffer, PBS, and blank. (C) XPS spectra at different stages of the synthesis (bare, PEI‐coated, Au‐seed‐functionalized, and Au‐coated). (D) Quantification of HSA adsorbed on the surface of the nanoparticles. Non‐specific adsorption on AuMags with and without BSA coating was determined. (E) Normalized Raman spectra of EpCAM–AuMagTags on a silicon wafer. The SERS peak of DTNB was at 1330 cm^−1^. (F) Schematic of PD‐L1 substrate preparation. (G) Atomic force microscopy. (H) Ra measurements at different stages of preparation (bare silicon wafer, sulfhydryl‐ functionalized surface [MPTMS], amine‐to‐sulfhydryl crosslinking [GMBS], antibody attachment [PD‐L1], and antifouling coating [BSA]). (I) X‐ray photoelectron spectra showing surface modifications. (J) Quantification of the amount of HSA adsorbed per unit area. Non‐specific adsorption on PD‐L1 substrate with and without BSA coating was determined.

For the selective capture of EpCAM^+^ EVs, AuMags were functionalized with EpCAM antibodies and 5,5'‐dithiobis‐(2‐nitrobenzoic acid) (DTNB, Ellman's reagent) as a Raman reporter (Figure 3a). To minimize non‐specific interactions, residual sites on the surface were blocked with bovine serum albumin (BSA). Antibody conjugation was performed in carbonate–bicarbonate buffer to mitigate interference from the nanoparticle surfactant layer. Phycoerythrin (PE)‐labeled EpCAM antibodies were conjugated to the AuMags under two different conditions (carbonate–bicarbonate buffer and PBS) to determine conjugation efficiency. Fluorescence intensities suggested more efficient antibody binding in carbonate–bicarbonate buffer compared to PBS (Figure 3b). X‐ray photoelectron spectroscopy was performed after each step of surface modification. The presence of the N 1s peak after coating the MNCs with PEI, and the presence of the characteristic Au 4f peak, confirmed the gold coating. Antibody immobilization on the AuMag surface was confirmed by the reduction in Au 4f binding energy (Figure 3c and Figure S2). This shift can be attributed to the coordination between the gold surface and electron‐donating groups in the antibody, consistent with previous study on protein–gold interactions [57]. To evaluate the effectiveness of BSA in blocking non‐specific adsorption, a protein adsorption test was performed using human serum albumin (HSA) (Figure 3d). A marked increase in HSA adsorption was observed in the absence of BSA (AuMags). Both BSA‐coated and DTNB‐tagged (AuMagTag) AuMags showed minimal non‐specific binding. To assess SERS tagging functionality, DTNB was conjugated to the surface of EpCAM‐conjugated AuMags (EpCAM–AuMags), and Raman spectra were recorded. EpCAM–AuMagTags exhibited distinct peaks at 1331 and 1557 cm^−1^, corresponding to the symmetric and asymmetric stretching modes of the nitro group, respectively. Signal intensities were enhanced compared to non‐tagged controls, confirming plasmonic amplification by the gold shell (Figure 3e).

An anti‐PD‐L1 antibody‐functionalized substrate (PD‐L1 substrate) was prepared by sequential modification—(3‐mercaptopropyl) trimethoxysilane (MPTMS) silanization, N‐γ‐maleimidobutyryl‐oxysuccinimide ester (GMBS) activation, anti‐PD‐L1 antibody immobilization, and BSA blocking—for the selective capture of PD‐L1‐positive (PD‐L1^+^) EVs and analysis of PD‐L1 expression on EVs (Figure 3f) [58]. Atomic force microscopy was used to visualize the changes in surface morphology at each modification step (Figure 3g). The bare silicon wafer exhibited a smooth surface, which became increasingly rough after MPTMS silanization and GMBS activation. After antibody conjugation, the surface exhibited a heterogeneous granular morphology. Subsequent BSA blocking further increased the surface roughness, indicating the formation of a dense protein layer. Quantitative analysis showed a stepwise increase in average surface roughness (Ra), facilitating the sequential assembly of the functional interface (Figure 3h). X‐ray photoelectron spectroscopy (XPS) provided evidence of stepwise surface functionalization. The emergence of a distinct S 2p peak following MPTMS silanization confirmed the presence of thiol groups on the substrate. The S 2p signal markedly decreased upon GMBS activation, accompanied by a shift in the C 1s spectral profile, consistent with thiol–maleimide coupling. Immobilization of the anti‐PD‐L1 antibody resulted in an increase in the N 1s peak and further modification of the C 1s spectral profile. BSA blocking further increased the N 1s and C 1s signals, confirming successful surface biofunctionalization (Figure 3i and Figure S3). The specificity of the substrate was validated by evaluating HSA adsorption. HSA adsorption was observed on both bare silicon wafers and the PD‐L1 antibody‐conjugated substrate surface prior to BSA blocking, indicating non‐specific interactions. After BSA blocking, HSA binding was markedly reduced, confirming that BSA blocking prevented non‐specific adsorption and increased surface specificity (Figure 3j). Collectively, these results confirm the successful stepwise synthesis of EpCAM–AuMagTags and the PD‐L1 substrate. Antibody conjugation, minimal non‐specific adsorption, and strong SERS signal amplification provide a robust platform for the detection of PD‐L1 expression on EpCAM^+^ EVs (EpCAM^+^ EV PD‐L1).

### 2.2 EpCAM^+^ EV PD‐L1 Assay

**FIGURE 4:**
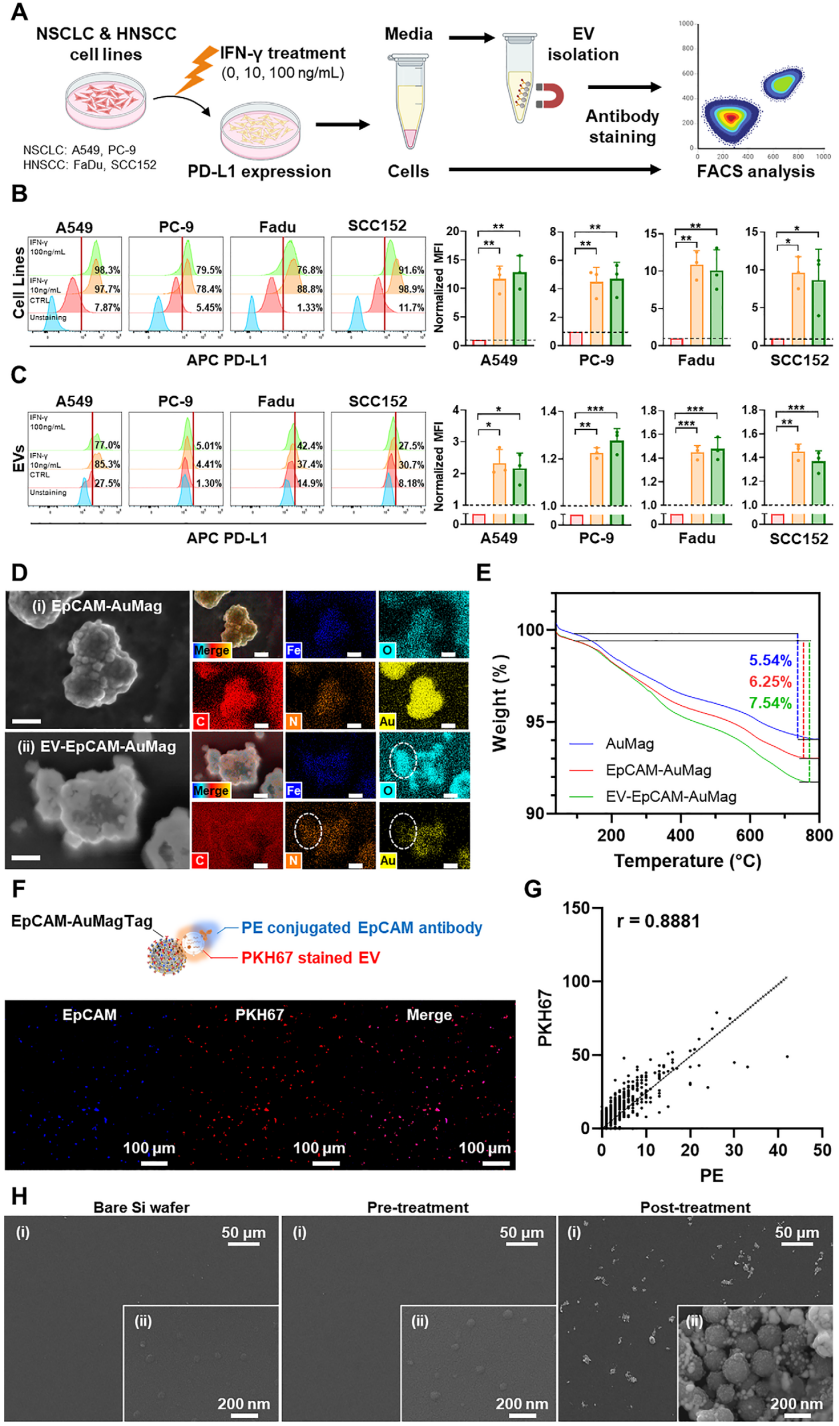
EpCAM^+^ EV PD‐L1 assay validation. (A) Schematic of IFN‐γ‐associated modulation of PD‐L1 expression in NSCLC and HNSCC cell lines in vitro. (B) Cell surface PD‐L1 expression. (C) Supernatant EpCAM^+^ EV PD‐L1 expression. Representative histograms are presented on the left (B, C), and mean fluorescence intensity (MFI) values were used to quantify PD‐L1 expression levels. (D) EDS mapping of EpCAM–AuMags (i) and EV–EpCAM–AuMags (ii). (E) Thermogravimetric curves of AuMags, EpCAM–AuMags, and EV–EpCAM–AuMags (scale bar: 200 µm). (F) Confocal fluorescence microscopy images showing the binding of EpCAM^+^ EVs to EpCAM–AuMagTags (PE–EpCAM antibody [blue]; PKH67‐stained EVs [red]). (G) Cytofluorogram with Pearson's correlation coefficient (r_p_) showing the correlation between PKH67 and PE fluorescence. (H) FE‐SEM images of the PD‐L1 substrate after treatment with EpCAM^+^/PD‐L1^+^ EVs captured by EpCAM–AuMagTags at low (i, ×500) and high (ii, ×100 000) magnifications.

To validate the SERS‐based EpCAM^+^ EV PD‐L1 assay, we examined the modulation of PD‐L1 expression in multiple NSCLC (A549 and PC‐9) and HNSCC (FaDu and SCC152) cell lines following IFN‐γ stimulation (Figure 4a). After IFN‐γ stimulation to induce PD‐L1 expression in vitro, EVs were isolated using CD63‐conjugated magnetic beads. In parallel, cells were detached from culture dishes using trypsin–EDTA to determine PD‐L1 expression on the cell surface. EVs and cells were stained with fluorescently labeled antibodies. Mean fluorescence intensity (MFI) was used to quantify the average PD‐L1 expression on the surface of cells and corresponding EpCAM^+^ EVs.

Flow cytometry revealed increased expression of PD‐L1 on the surface of all cell lines following IFN‐γ stimulation (Figure 4b). A comparable increase in PD‐L1 levels was observed in EVs isolated from cell culture supernatant (Figure 4c). These results confirm that EVs released from PD‐L1‐expressing cells reflect biologically relevant PD‐L1 expression, thereby supporting their application in a SERS‐based EpCAM^+^ EV PD‐L1 assay.

EVs isolated from NSCLC and HNSCC cell lines were characterized by western blotting, nanoparticle tracking analysis (NTA), and TEM. Western blot analysis confirmed the presence of the EV‐associated marker TSG101 (i) in EVs derived from all tested cell lines regardless of IFN‐γ treatment, whereas the negative marker calnexin (ii) was not detected in any EV samples, supporting the absence of major intracellular contaminants (Figure S4a). NTA analysis showed that the isolated EVs exhibited a size distribution consistent with small extracellular vesicles (approximately 75–139 nm depending on the cell line) and particle concentrations ranging from 0.72 to 1.94 × 10^10^ particles/mL (Figure S4b). No significant differences in EV size distribution were observed following IFN‐γ treatment. TEM analysis further confirmed the presence of vesicles with characteristic spherical nanoscale morphology, without noticeable morphological changes following IFN‐γ treatment (Figure S4c).

Field‐emission scanning electron microscopy (FE‐SEM) was performed to further demonstrate the capture of EpCAM^+^ EVs by EpCAM–AuMagTags. Following incubation with IFN‐γ‐treated SCC152 cell culture medium, the surface of the particles exhibited distinct morphological changes and accumulation of organic matter (Figure 4d and Figure S5). EDS mapping showed a decrease in the Au signal with a concomitant increase in the N and O signals, consistent with protein binding. Thermogravimetric analysis further confirmed the accumulation of organic matter on the particle surface (Figure 4e). To identify the captured material, the separated particles were stained with PE–EpCAM antibodies and the lipophilic membrane dye PKH67 (Figure 4f) [59]. Fluorescence microscopy showed strong signal colocalization, confirming the expression of EpCAM and the presence of a lipid bilayer, hallmarks of EVs. Quantitative colocalization analysis supported these findings, yielding a Pearson's correlation coefficient (r_p_) of 0.8881 (Figure 4g). Collectively, these results confirm that the captured material was EpCAM^+^ EVs, rather than non‐vesicular particles or non‐specific protein aggregates. To demonstrate the suitability of the PD‐L1 substrate for use in the SERS‐based EpCAM^+^ EV PD‐L1 assay, EV‐bound EpCAM–AuMagTags (EV–EpCAM–AuMagTags) were prepared using conditioned media derived from IFN‐γ‐treated SCC152 cells and subsequently applied to the substrate. FE‐SEM analysis confirmed the binding of EpCAM^+^/PD‐L1^+^ EVs to the substrate surface (Figure 4h). At low magnification (i), the bare silicon wafer and pre‐treatment surfaces exhibited no discernible features. In contrast, post‐treatment images revealed extensive deposition of particulate matter on the substrate. High‐magnification images (ii) further revealed distinct nanostructures, confirming the successful PD‐L1^+^ EV‐mediated immobilization of EV–EpCAM–AuMagTags.

The analytical reliability of the platform was validated by assessing EV capture efficiency, interfacial stability, and assay reproducibility (Figure S6). EpCAM–AuMagTag exhibited EV capture efficiency comparable to that of EpCAM–AuMag, indicating that the tag integration does not compromise isolation performance (Figure S6a). The EpCAM antibody‐associated fluorescence of EpCAM–AuMagTag retained stable in plasma for up to 6 h, with no significant signal loss, exceeding the duration required for the process (1 h) (Figure S6b). In addition, the platform demonstrated high reproducibility, with coefficients of variation (CV) within ∼10% across independent batches and different experimental days (Figure S6c), supporting its reliability for consistent EV‐based analyses.

**FIGURE 5:**
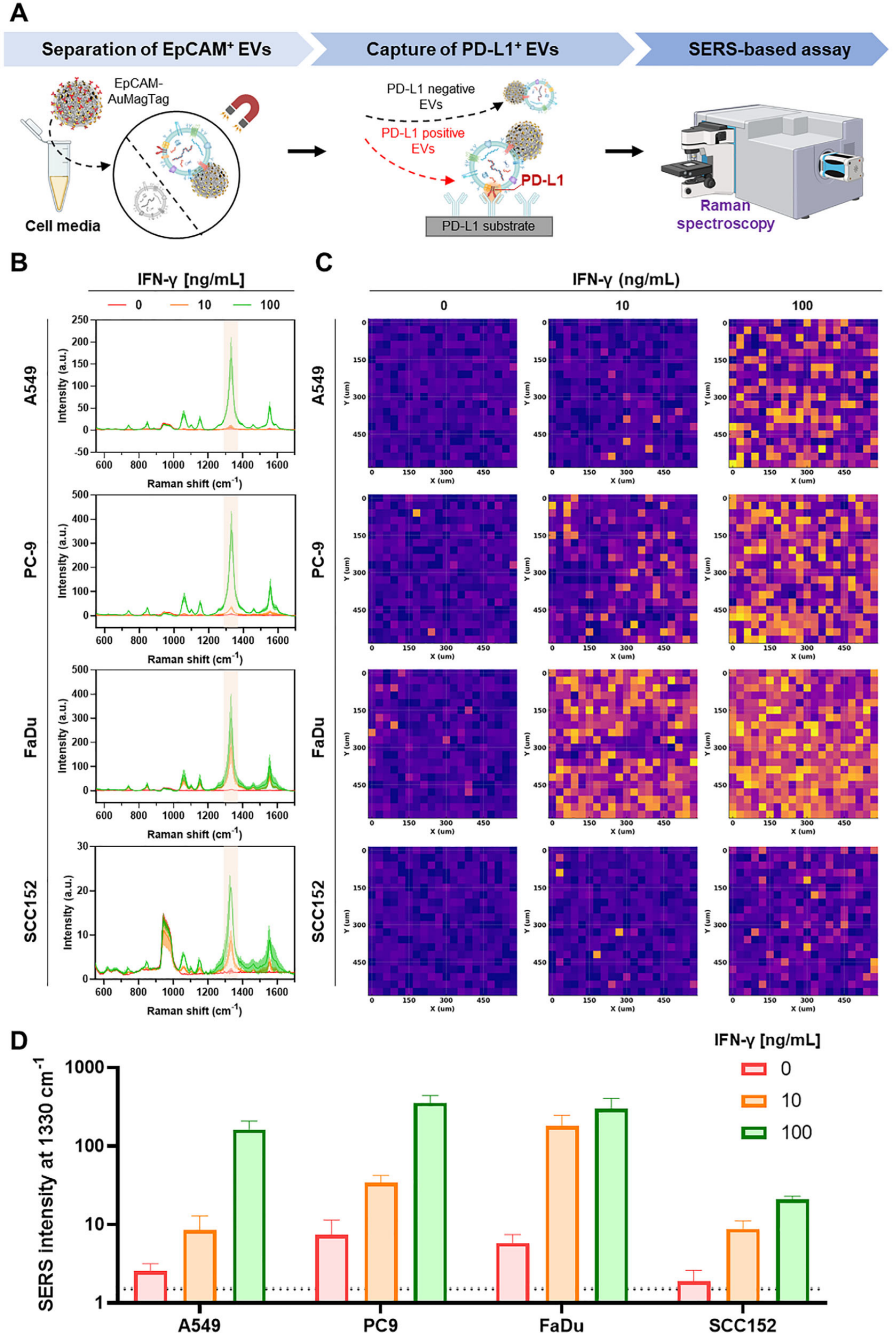
Evaluation of EpCAM^+^ EV PD‐L1 assay using cell supernatants. (A) Schematic illustration of the SERS‐based EpCAM^+^ EV PD‐L1 assay workflow applied to cell culture supernatants from A549, PC‐9, FaDu, and SCC152 cells. (B) Average Raman spectra obtained from conditioned media of the four cell lines treated with increasing concentrations of IFN‐γ (0, 10, and 100 ng/mL). (C) Heatmap of the characteristic SERS peak at 1330 cm^−1^, quantified from the spectra shown in (B). (D) Quantification of the average intensity of the characteristic peak (averaged over 400 mapping points per measurement, *n* = 3 independent measurements).

SERS analysis was performed to evaluate the performance of the EpCAM^+^ EV PD‐L1 assay using EpCAM^+^ EVs isolated from the conditioned media of A549, PC‐9, FaDu, and SCC152 cell lines treated with increasing concentrations of IFN‐γ (Figure 5a). The workflow involved the selective enrichment of EpCAM^+^ EVs using EpCAM–AuMagTags, followed by immunoaffinity capture of PD‐L1^+^ EVs on the PD‐L1 substrate. Raman spectra were acquired for the surface‐bound complexes, enabling ultrasensitive quantification of PD‐L1 expression on EpCAM^+^ EVs. The Raman spectra exhibited distinct dose‐dependent changes in response to IFN‐γ stimulation in all four cell lines (Figure 5b,c). The average intensity of the characteristic SERS peak at 1,330 cm^−1^ (averaged over 400 mapping points per measurement, n = 3 independent measurements) increased proportionally with IFN‐γ concentrations, closely mirroring the levels of PD‐L1 determined by flow cytometry (Figure 5d). Negative control (NC, EpCAM–AuMagTags only) and untreated substrate (NT) signals did not deviate from baseline and were used to determine the reference threshold (dashed line). EDS mapping showed that when EpCAM and PD‐L1 were abundantly expressed, SERS signals were uniformly distributed across a broad spatial domain with high reproducibility (Figure S7). In addition, to validate PD‐L1–specific capture, an isotype antibody–coated substrate was used as a control. As shown in Figure S8a, the isotype control exhibited negligible SERS signal, and quantitative analysis (Figure S8b) showed intensity levels comparable to the NC and NT. These results indicate minimal nonspecific adsorption and confirm PD‐L1–specific interactions, supporting the reliability of the SERS platform for sensitive and accurate detection of EpCAM^+^ EV PD‐L1. Magnetic pre‐enrichment removed background contaminants and isolated EpCAM^+^ EVs, thereby enhancing signal specificity and minimizing non‐specific interference. Collectively, these results show that the SERS platform is robust in detecting PD‐L1 expression and highlights its potential for clinical application.

### 2.3 EpCAM^+^ EV PD‐L1 Dynamics

Using the aforementioned SERS‐based EpCAM^+^ EV PD‐L1 assay, we conducted a double‐blind analysis of 213 specimens (140 NSCLC and 73 HNSCC). For each sample, the average SERS signal was calculated from 400 measurement points to determine the abundance of EpCAM^+^/PD‐L1^+^ EVs (Figure S9). Given the large dataset, the complete set of images is provided as Figures S10–S20. Although the overall SERS signal intensity was lower than that observed in the cell lines, the presence of a discernible DTNB spectral signature allowed reliable quantification based on the peak at 1330 cm^−1^. This attenuation reflects the inherently low concentration of EVs isolated from plasma, which may account for the difficulty in analyzing such samples using fluorescence‐based assays such as flow cytometry. We also investigated the association between EpCAM^+^ EV PD‐L1 levels and treatment response in patients receiving anti‐PD‐(L)1 immunotherapy. Specifically, we analyzed EpCAM^+^ EV PD‐L1 levels at baseline (immediately before treatment) and on‐treatment (14 days after the start of treatment) (Table S1) in patients with advanced NSCLC (*n* = 140) or HNSCC (*n* = 73).

**FIGURE 6:**
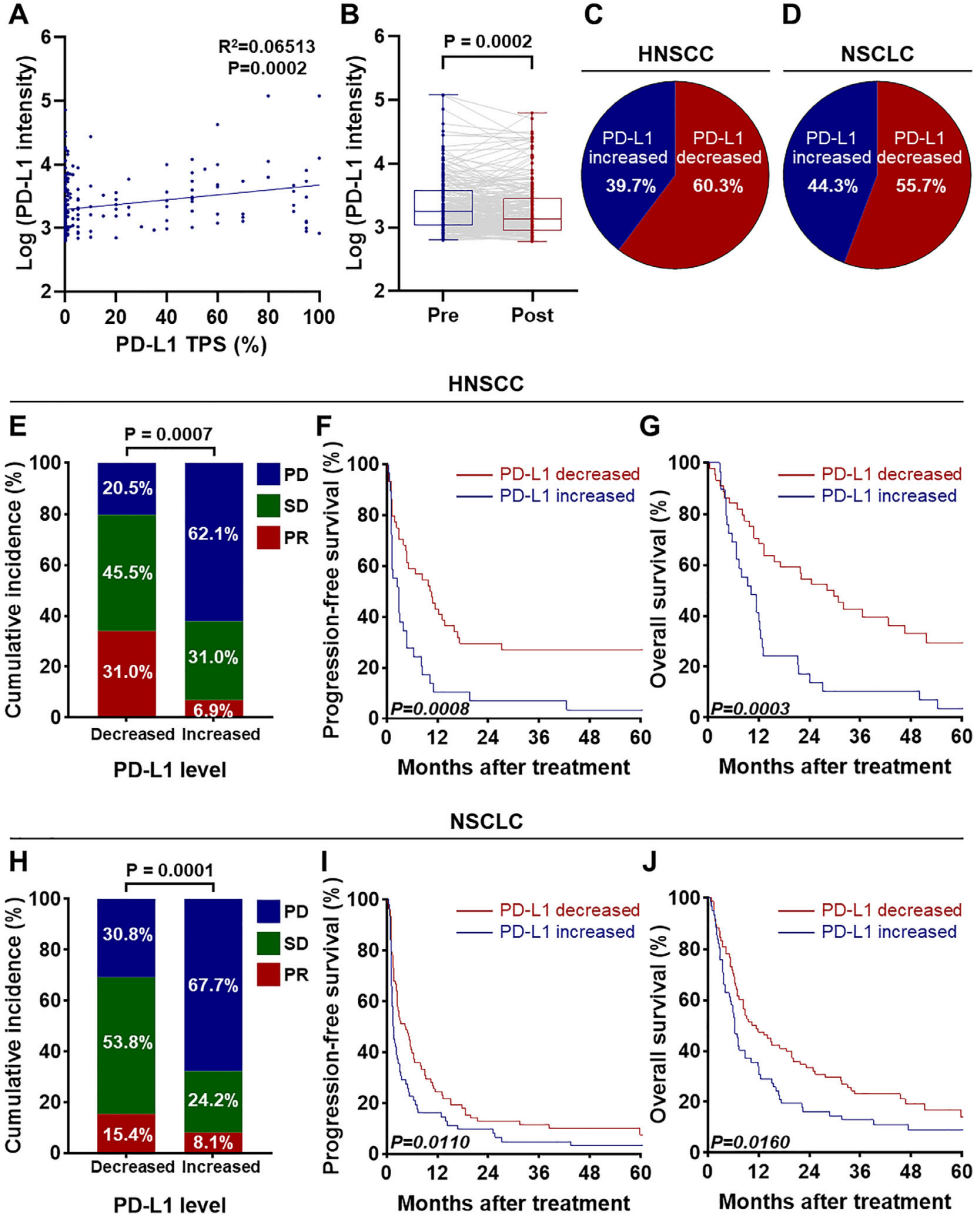
EpCAM^+^ EV PD‐L1 dynamics and treatment outcomes. (A) Correlation between PD‐L1 expression in tumor tissues and EpCAM^+^ EV PD‐L1 expression in blood. (B) Changes in EV PD‐L1 expression in blood upon treatment with PD‐(L)1 inhibitors. (C) Distribution of patients with HNSCC with increased (blue) or decreased (red) EpCAM^+^ EV PD‐L1 expression after treatment. (D) Distribution of patients with NSCLC with increased (blue) or decreased (red) EpCAM^+^ EV PD‐L1 expression after treatment. (E) Best response to PD‐(L)1 inhibitors according to changes in EpCAM^+^ EV PD‐L1 expression in patients with HNSCC. (F) Progression‐free survival after treatment with PD‐(L)1 inhibitors according to changes in EpCAM^+^ EV PD‐L1 expression in patients with HNSCC. (G) Overall survival after treatment with PD‐(L)1 inhibitors according to changes in EpCAM^+^ EV PD‐L1 expression in patients with HNSCC. (H) Best response to PD‐(L)1 inhibitors according to changes in EpCAM^+^ EV PD‐L1 expression in patients with NSCLC. (I) Progression‐free survival after treatment with PD‐(L)1 inhibitors according to changes in EpCAM^+^ EV PD‐L1 expression in patients with NSCLC. (J) Overall survival after treatment with PD‐(L)1 inhibitors according to changes in EpCAM^+^ EV PD‐L1 expression in patients with NSCLC.

In 193 patients with available PD‐L1 immunohistochemistry data, a weak positive correlation was observed between tumor PD‐L1 expression and baseline EpCAM^+^ EV PD‐L1 levels (R^2^ = 0.06513, *p* = 0.0002) (Figure 6a). This modest association may have resulted from tumor heterogeneity, sampling bias inherent to tissue biopsy, and temporal discordance between tissue acquisition and blood sampling, given the dynamic nature of PD‐L1 expression.

Next, we assessed whether dynamic changes in EpCAM^+^ EV PD‐L1 expression relative to baseline could predict the clinical outcome of NSCLC and HNSCC. PD‐(L)1 inhibitor treatment led to a significant reduction in EpCAM^+^ EV PD‐L1 levels (Figure 6b). PD‐L1 expression was reduced in 55.7% and 60.3% of patients with NSCLC and HNSCC, respectively (Figure 6c,d). Among patients with HNSCC, those with reduced PD‐L1 expression had a higher objective response rate than those without (31.0% vs. 6.9%, respectively) (Figure 6e). These findings highlight the clinical significance of dynamic monitoring of EpCAM^+^ EV PD‐L1 expression, suggesting that early reductions in circulating PD‐L1 levels may reflect a favorable immunologic response and better therapeutic efficacy [29, 60]. This reduction was associated with improved progression‐free and overall survival (Figure 6f,g). Baseline EpCAM^+^ EV PD‐L1 levels, however, were not predictive of outcome (Figure S21a–c). These results underscore the prognostic value of monitoring on‐treatment dynamics rather than relying solely on static baseline measurements, thereby supporting the clinical utility of dynamic changes in EpCAM^+^ EV PD‐L1 expression as a minimally invasive biomarker of treatment response. Comparable results were obtained in patients with NSCLC (Figure 6h–j and Figure S21d–f). Importantly, multivariable analysis adjusting for age, sex, smoking status, line of treatment, and tumor PD‐L1 expression demonstrated that reductions in EpCAM^+^ EV PD‐L1 levels independently predicted improved PFS and OS in both HNSCC (Table S2) and NSCLC (Table S3). Additionally, dynamic changes in EpCAM^+^ EV PD‐L1 expression was associated with durable clinical benefit in both HNSCC and NSCLC, whereas tumor PD‐L1 expression during PD‐(L)1 inhibitor treatment was associated with clinical benefit in only NSCLC during PD‐(L)1 inhibitor treatment (Figure S22a–d). The consistency of these findings across both cancer types strengthens the generalizability of this approach and suggests a shared mechanism underlying PD‐L1 modulation during immune checkpoint inhibitor treatment. To further support these findings, we conducted a double‐blind analysis of 69 specimens from patients receiving cytotoxic chemotherapy. In this cohort, reductions in EpCAM^+^ EV PD‐L1 was not associated with favorable outcomes (Figure S23a–e), suggesting that dynamic changes in EpCAM^+^ EV PD‐L1 may represent a treatment‐specific biomarker for PD‐(L)1 blockade rather than a nonspecific surrogate of tumor burden.

Collectively, these results show that early changes in EpCAM^+^ EV PD‐L1 (measured within 2 weeks of treatment initiation) can predict treatment response to PD‐(L)1 inhibitors in patients with advanced NSCLC and HNSCC. The integration of the SERS‐based EpCAM^+^ EV PD‐L1 assay enabled the rapid, ultrasensitive, and non‐invasive measurement of PD‐L1 kinetics using small volumes of plasma, overcoming key limitations of conventional tissue‐based and bulk EV analysis. Importantly, early reductions in EpCAM^+^ EV PD‐L1 expression following PD‐(L)1 blockade were associated with improved objective response rates and survival in patients with NSCLC and HNSCC, highlighting the potential of this approach for real‐time therapeutic monitoring and patient stratification. While further prospective studies of larger, more heterogeneous populations are warranted, these findings establish a basis for the clinical translation of EV‐based immune monitoring and open new avenues for precision oncology guided by circulating biomarkers.

## 3 Discussion

In the present study, we developed a SERS platform for dynamic monitoring of EpCAM^+^ EV PD‐L1 and demonstrated its value as a clinically useful biomarker. Unlike conventional tissue‐based assays that offer only a static snapshot of PD‐L1 expression, the SERS platform enables real‐time monitoring of treatment‐induced changes in circulating EpCAM^+^ EV PD‐L1, offering a more sensitive and temporally relevant measure of therapeutic response. Dynamic monitoring of EpCAM^+^ EV PD‐L1 proved superior to baseline tissue‐based assessment of PD‐L1 expression for predicting treatment response. While tissue PD‐L1 expression showed only a weak correlation with baseline EpCAM^+^ EV PD‐L1 levels (R^2^ = 0.06513), dynamic changes in circulating EpCAM^+^ EV PD‐L1 within 14 days of treatment initiation strongly predicted the objective response rate and long‐term survival. This finding addresses a key limitation of tissue‐based biomarkers, which offer only a static snapshot of tumor biology and may not capture the evolving immune landscape during treatment. In contrast, circulating EpCAM^+^ EV PD‐L1 provides real‐time insight into systemic tumor–immune dynamics, allowing for a more timely and accurate assessment of therapeutic response.

The success of our approach lies in the strategic selection of the well‐established epithelial marker EpCAM—which is highly expressed in various cancers, making it a reliable marker for isolating tumor‐derived EVs—as the target. Its surface localization and stable expression offer a practical advantage for antibody‐based enrichment. EpCAM‐based enrichment enhanced the tumor specificity of the SERS‐based EV PD‐L1 assay by selectively capturing epithelial tumor‐derived EVs from the heterogeneous pool of circulating EVs. This approach amplified tumor‐associated signals, while reducing background noise from immune and non‐tumor cells. Magnetic pre‐enrichment enabled the detection of low‐abundance targets that would otherwise remain undetected in conventional assays, as demonstrated by our ability to quantify EpCAM^+^ EV PD‐L1 in plasma.

Besides the preclinical compatibility of the SERS platform, we also explored the clinical relevance of EpCAM^+^ EV PD‐L1 in patients treated with immune checkpoint inhibitors. Early reductions in EpCAM^+^ EV PD‐L1 were associated with improved survival in patients with NSCLC or HNSCC treated with anti‐PD‐(L)1 therapy. Reproducibility across different cancers highlights the translational potential of EpCAM^+^ EV PD‐L1 as a universal biomarker. Early reductions in EpCAM^+^ EV PD‐L1 expression during immunotherapy predicted favorable outcomes in 55.7% and 60.3% of patients with NSCLC and HNSCC, respectively; this suggests a shared mechanism underlying EpCAM^+^ EV PD‐L1 modulation during immune checkpoint inhibitor therapy. These results support the development of integrated biomarker strategies for epithelial cancers, potentially streamlining clinical implementation and accelerating the adoption of personalized immunotherapy monitoring.

Beyond its biological relevance, the EpCAM^+^ EV PD‐L1 platform may offer several advantages for clinical decision‐making. First, dynamic changes in EpCAM^+^ EV PD‐L1 could enable early identification of treatment response or resistance, potentially preceding radiographic evaluation. This may facilitate more timely treatment modifications, such as continuation of effective therapy or early switching or intensification in non‐responders. Second, as a minimally invasive liquid biopsy approach, this platform allows longitudinal monitoring of tumor dynamics throughout the course of immunotherapy, which may be particularly valuable in settings where repeated tissue sampling is not feasible. Third, by enriching tumor‐derived exosomes, this approach may provide a more tumor‐specific signal compared to conventional circulating biomarkers, supporting its potential role in guiding personalized immunotherapy strategies.

Nevertheless, the SERS platform has some limitations that warrant further consideration. Although EpCAM exhibits strong lineage specificity for epithelial cells compared with CD63 and CD81 (Figure S24), EpCAM expression varies across cancers and may be reduced in dedifferentiated tumors or those undergoing epithelial–to–mesenchymal transition. This approach may underrepresent contributions from stromal‐ or immune cell‐derived EVs that could carry biologically relevant signals. Future studies should investigate these potential biases and consider complementary enrichment strategies for more comprehensive EV profiling. Further analytical development and standardization are critical for the integration of the SERS platform into routine oncology practice. Prospective clinical trials incorporating non‐invasive monitoring of EpCAM^+^ EV PD‐L1 expression could support adaptive treatment strategies beyond retrospective validation, ultimately guiding patient stratification and optimizing therapeutic decision‐making in precision immuno‐oncology.

In conclusion, we demonstrated that EpCAM^+^ EV PD‐L1 expression can serve as a clinically relevant biomarker. This approach provides a robust technological foundation for monitoring and predicting responses to immunotherapy based on next‐generation liquid biopsy using the SERS platform. Early identification of non‐responders to standard treatment through EV‐based biomarker detection could guide timely treatment modifications, additional combination therapy, or initiation of alternative treatment. Prospective adaptive clinical trials incorporating real‐time monitoring of EpCAM^+^ EV PD‐L1 could improve treatment and outcomes, while reducing unnecessary toxicity.

## 4 Experimental Section

### 4.1 Materials

Iron (III) chloride, sodium citrate, sodium acetate, ethylene glycol, diethylene glycol, branched PEI (average Mw = 25 k), gold (III) chloride trihydrate, tetrakis (hydroxymethyl) phosphonium chloride, polyvinylpyrrolidone (average Mw = 55 k), L‐ascorbic acid, human serum, carbonate–bicarbonate buffer, BSA, HSA, sodium dodecyl sulfate, MPTMS, the PKH67 Green Fluorescent Cell Linker Kit, and Phosphotungstic Acid were purchased from Sigma–Aldrich (St. Louis, MO, USA). PBS (1×) was purchased from Welgene (Gyeongsan, Republic of Korea). Roswell Park Memorial Institute (RPMI)‐1640 medium was purchased from Corning (Corning, NY, USA). Foetal bovine serum (FBS), penicillin–streptomycin, trypsin–EDTA (0.25%), and no phenol red were purchased from Gibco (Grand Island, NY, USA). Single donor human red blood cells were purchased from Innovative Research (Novi, MI, USA). CD326 (EpCAM) Monoclonal Antibody (1B7) (eBioscience; Cat. No. 14‐9326‐95), PE‐conjugated CD326 (EpCAM) Monoclonal Antibody (1B7) (eBioscience; Cat. No. 12‐9326‐42), DTNB, the Micro BCA Protein Assay Kit, Sulfo‐GMBS, human IFN‐γ recombinant protein (Cat. No. 300‐02), Dynabeads Streptavidin T1, and the Live/Dead Fixable Near‐IR Dead Cell Stain Kit, Goat anti‐Rabbit IgG (H+L) Secondary Antibody (Cat. No. 31460), Pierce ECL Western Blotting Substrate, eFluor 660‐conjugated CD326 (EpCAM) Monoclonal Antibody (1B7) (eBioscience; Cat. No. 50‐9326‐42), and Mouse IgG1 kappa Isotype Control (P3.6.2.8.1) (eBioscience; Cat. No. 14‐4714‐82) were purchased from Thermo Fisher Scientific (Waltham, MA, USA). The silicon wafer (4″) was purchased from Taewon Scientific (Seoul, Republic of Korea). Monoclonal mouse anti‐human PD‐L1 antibody (22C3) (Cat. No. M365329‐2) and the PD‐L1 IHC 22C3 and 28‐8 PharmDx assays were purchased from Agilent Technologies (Santa Clara, CA, USA). The PD‐L1 (SP142 and SP263) assays were purchased from Ventana Medical Systems (Tuscon, AZ, USA). Biotin Anti‐CD63 antibody (MEM‐259) (ab134331), Anti‐TSG101 antibody (EPR7130(B)) (ab125011), and Anti‐calnexin antibody (ab22595) was purchased from Abcam (Cambridge, UK). PE anti‐human CD326 (EpCAM) (Cat. No. 324206) antibody was purchased from BioLegend (San Diego, CA, USA). Allophycocyamin (APC) mouse anti‐human CD274 (Cat. No. 563741) antibody was purchased from BD Biosciences (San José, CA, USA). Exo‐spin^TM^ exosome purification kit was purchased from Cell Guidance Systems (Cambridge, UK). Radioimmunoprecipitation assay (RIPA) buffer were purchased from LPS Solution (Daegu, Republic of Korea). Skim milk was purchased from BD Difco (Sparks, MD, USA).

### 4.2 Synthesis and Characterization of AuMags

MNCs were synthesized using a solvothermal reaction as described previously [54, 55, 56]. Briefly, iron (III) chloride, sodium acetate, and sodium citrate were dissolved in a co‐solvent of ethylene glycol and diethylene glycol. The mixture was stirred vigorously and sonicated, and then sealed in an acid digestion vessel with a polytetrafluoroethylene liner. The reactor was autoclaved for 12 h at 220°C and cooled to 25°C ± 2°C. The synthesized MNCs were purified by washing with ethanol and distilled water. MNCs (5 mL; 1 mg mL^−1^) were redispersed in distilled water, dried by magnetic separation, and mixed with PEI (5 mL; 50 mg mL^−1^). The mixture was stirred vigorously for 6 h. MNC@PEIs were washed with distilled water to remove unbound PEI.

AuMags were synthesized using a seed‐mediated growth method. To prepare the gold seed solution, sodium hydroxide (0.5 mL; 1 M) and tetrakis (hydroxymethyl) phosphonium chloride (12 µL) were mixed with distilled water (45 mL). After 5 min, gold (III) chloride trihydrate (2 mL; 1 wt.%) was rapidly added and the solution was stirred for 1 h at 25°C ± 2°C. The gold seed solution (5 mL) was mixed with solvent‐removed MNC@PEIs for 6 h at 25°C ± 2°C. MNC@PEI‐Seeds were washed with and redispersed in PBS (5 mL). MNC@PEI‐Seeds (0.2 mL) were subsequently mixed with polyvinylpyrrolidone (1 mL; 0.25 wt.%) and gold (III) chloride trihydrate (4 mL; 0.1 wt%). The mixture was stirred vigorously and sonicated. Ascorbic acid (0.1 mL; 0.1 M) was added and the mixture was immediately vortexed and sonicated three times alternately for 1 min each. AuMags were washed with and redispersed in PBS (0.2 mL).

The morphology and elemental composition of the MNCs, MNC@PEI‐Seeds, and AuMags were examined using TEM (JEM‐F200; JEOL, Tokyo, Japan) equipped with EDS. Elemental mapping of Fe, O, C, N, and Au was performed to confirm the spatial distribution of the particles. The hydrodynamic size and zeta potential at each stage of the synthesis were measured by dynamic light scattering (ELSZ‐2000ZS; Otsuka Electronics, Osaka, Japan). The magnetic hysteresis loops and saturation magnetization were assessed in dried samples at 298 K using a vibrating sample magnetometer (Model‐7300; Lakeshore, Carson, CA, USA). Crystalline structures were determined by X‐ray diffraction using an X‐ray diffractometer (Ultima IV; Rigaku, Tokyo, Japan). The formation of gold‐coated MNC@PEIs was confirmed by UV–vis absorbance spectra using a microplate reader (Spectra Max i3x; Molecular Devices, San José, CA, USA). To determine the magnetic separability of AuMags, 0.2 mL of AuMags was added to 1 mL of each of the following: PBS, RPMI‐1640 supplemented with 10% FBS and 1% penicillin–streptomycin, serum, and red blood cells. The process was documented by sequential imaging: (i) initial solution, (ii) dispersed particles, (iii) magnetically isolated particles, and (iv) particles redispersed in distilled water (0.2 mL).

### 4.3 Preparation of EpCAM–AuMagTags

AuMags were redispersed in carbonate/bicarbonate buffer (1 mL; pH ∼9.6). EpCAM antibody (5 µg) was added and the mixture was incubated for 12 h at 4°C with gentle shaking. EpCAM‐conjugated AuMags were washed with PBS to remove unbound antibody. The Raman dye DTNB (10 µL; 1 mm) was added and BSA (1 mL; 0.1 wt.%) was used to block non‐specific binding sites. The mixture was incubated for 12 h at 4°C followed by thorough washing. EpCAM–AuMagTags were redispersed in PBS (0.2 mL) and stored at 4°C.

PE–EpCAM antibodies were used following the same procedure as that for EpCAM antibodies to evaluate conjugation efficacy. For comparison, two controls were prepared: AuMags and AuMags prepared in PBS instead of carbonate/bicarbonate buffer. Equal volumes (0.2 mL) of AuMags suspensions were conjugated with 5 µg of PE‐EpCAM antibodies. After conjugation, particles were washed using magnetic separation to remove unbound antibodies, and fluorescence was measured from the particle‐bound fraction using a microplate reader (Spectra Max i3x). XPS (K‐Alpha; Thermo Fisher Scientific) was used to analyze the elemental composition (Fe, O, N, C, and Au composition) of the particles at each synthesis step.

A protein adsorption test was performed using HSA to evaluate the effectiveness of BSA in blocking non‐specific adsorption) [59]. HSA (45 mg mL^−1^) was added to unmodified, BSA‐coated, and EpCAM‐conjugated AuMags. Each sample was incubated with HSA for 1 h at 25°C ± 2°C on a shaker, followed by triple washing with PBS to remove unbound proteins. The particles were incubated in PBS containing sodium dodecyl sulfate (5% w/v) for 5 min to detach the adsorbed protein. The supernatant was collected by magnetic separation and diluted in PBS (350 µL). The protein content of the supernatant was quantified in triplicate using a Micro BCA Protein Assay Kit, following the manufacturer's instructions.

To confirm successful dye conjugation, EpCAM–AuMagTags (with DTNB) and EpCAM–AuMags (without DTNB) were drop‐cast onto 1.5 × 1.5 mm silicon wafers and dried under ambient conditions. Raman spectra were measured at nine points using a Raman spectrometer (LabRAM ARAMIS, Horiba, Kyoto, Japan) equipped with a 100× objective lens (Nikon; numeral aperture, 0.95) and an Nd:YAG laser (λ = 633 nm). The laser power at the sample surface was ∼4.5 mW (as measured at the objective). The acquisition time was set to 1s per point. Spectra were processed in MATLAB (MathWorks Inc., Natick, MA, USA) with Savitzky–Golay baseline correction implemented in MATLAB (MathWorks Inc.) [58].

### 4.4 Preparation of the PD‐L1 Substrate

The antibody‐functionalized substrate was prepared as previously described [56]. Briefly, a silicon wafer was diced into 1.5 × 1.5 mm squares, rinsed with acetone, and treated with oxygen plasma for 2 min at a radio frequency power of 100 W to activate the surface. The wafer pieces were incubated in an MPTMS–ethanol (5% w/v) solution for 1 h at 25°C ± 2°C with gentle shaking. After silanization, the substrates were rinsed sequentially with ethanol and distilled water. The MPTMS‐functionalized wafer pieces were incubated in an aqueous solution of sulfo‐GMBS (0.38 mg mL^−1^) for 30 min and washed with PBS. The wafer pieces were then incubated with anti‐PD‐L1 antibodies diluted in PBS (0.1 mg mL^−1^) for 1 h at 25°C ± 2°C. After antibody immobilization, the surfaces were rinsed with PBS. BSA (1%) in PBS was used to block non‐specific binding sites. Surface characterization was performed after every step of surface modification and antibody immobilization. Topographical analyses were performed using atomic force microscopy (NX‐10; Park Systems, Suwon, Republic of Korea). XPS (K‐Alpha) was used to analyze the elemental—O, N, C, S, and Si— composition of the wafer surface. A protein adsorption test was performed using HSA to evaluate the effectiveness of BSA in blocking non‐specific adsorption, as described above for EpCAM–AuMags.

### 4.5 Cell Culture

NSCLC cell lines (A549 and PC‐9) were kindly gifted by Prof. Sang‐Jun Ha (Yonsei University, College of Life Science & Biotechnology, Department of Biochemistry, Seoul, Republic of Korea) and FaDu cell line was kindly gifted by Prof. Dahee Kim (Yonsei University, College of Medicine, Department of Otorhinolaryngology, Seoul, Republic of Korea). SCC152 cell line was purchased from ATCC (CRL‐3240). All cell lines were cultured in RPMI‐1640 medium supplemented with 10% FBS and 1% penicillin–streptomycin at 37°C in a humidified incubator with 5% CO_2_. Cells were sub‐cultured every 2–3 days after reaching ∼80% confluency.

### 4.6 IFN‐γ Stimulation

Cells were pre‐cultured for 1 week under standard conditions. The culture medium was removed, the cells were gently washed with PBS, IFN‐γ was added at a final concentration of 10 or 100 ng mL^−1^ in RPMI‐1640 medium supplemented with 2% FBS and 1% penicillin–streptomycin, and the cells were incubated for 72 h under standard conditions.

### 4.7 Multicolor Flow Cytometry

Cells were detached from the culture dishes using 0.25% trypsin and collected by centrifugation. In parallel, Extracellular vesicles (EVs) were isolated from the culture medium using CD63 antibody‐conjugated magnetic beads (Beads). Biotinylated CD63 antibody was conjugated to Dynabeads Streptavidin T1, according to the manufacturer's instructions. The cells and Bead‐bound EVs were washed with cold flow cytometry buffer (PBS supplemented with 2% FBS). Cells were stained with PE–EpCAM (1:50) and APC–PD‐L1 (1:50) antibodies using the Live/Dead Fixable Near‐IR Dead Cell Stain Kit. Bead‐bound EVs were stained with PE–EpCAM (1:50), and APC–PD‐L1 (1:50) antibodies in flow cytometry buffer. Staining was performed for 20 min at 4°C in the dark. After staining, samples were washed three times with cold flow cytometry buffer. Multicolor flow cytometric analysis was performed using a CytoFLEX LX flow cytometer (Beckman Coulter, Brea, CA, USA). Both cells and EVs were gated based on EpCAM positivity and analyzed for PD‐L1 expression. The data were analyzed using FlowJo software (version 10.10.0) (Tree Star Inc., Ashland, OR, USA).

### 4.8 EV Characterization

EVs were isolated from cell culture supernatants using the Exo‐spin exosome purification kit according to the manufacturer's instructions. For protein characterization, isolated EVs were lysed in radioimmunoprecipitation assay (RIPA) buffer containing a protease inhibitor cocktail, and protein concentrations were determined using a bicinchoninic acid (BCA) assay. Proteins were separated by sodium dodecyl sulfate‐polyacrylamide gel electrophoresis (SDS‐PAGE) at 80 V for 150 min and transferred onto polyvinylidene difluoride (PVDF) membranes at 80 V for 90 min. The membranes were blocked with 5% skim milk in TBS‐T buffer (20 mm Tris‐HCl, pH 7.6, 150 mm NaCl, and 0.1% Tween 20) for 1 h at 25°C ± 2°C. To assess EV purity and potential cellular contamination, the PVDF membranes were incubated overnight at 4°C with primary antibodies against the EV marker TSG101 and the negative markers calnexin. This was followed by incubation with HRP‐conjugated anti‐rabbit secondary antibodies for 2 h at 25°C ± 2°C. Protein bands were visualized using Pierce ECL Western Blotting Substrate and imaged using a ChemiDoc MP imaging system (Bio‐Rad Laboratories, Hercules, CA, USA).

The size distribution and particle concentration of EVs were analyzed by nanoparticle tracking analysis (NTA) using a NanoSight Pro (Malvern Panalytical, Malvern, UK). EV morphology was further examined by TEM (JEM‐ARM200F, JEOL, Tokyo, Japan). For TEM analysis, EV samples were negatively stained with 1% phosphotungstic acid solution prior to imaging.

### 4.9 SERS Platform

To evaluate the effectiveness of the SERS platform in detecting PD‐L1 expression in EpCAM^+^ EVs, the capture of tumor‐derived EVs by EpCAM–AuMagTags was assessed using SCC152 cell culture supernatant. EpCAM–AuMagTags (20 µL) were incubated with IFN‐γ‐treated (100 ng mL^−1^; 200 µL) culture medium for 1 h at 37°C with gentle shaking. The particles were collected by magnetic separation and washed three times with PBS. The morphology and elemental composition of the particles at each stage of the synthesis were examined using FE‐SEM (JSM‐IT800SHL; JEOL) equipped with EDS. Elemental mapping of Fe, O, C, N, and Au was performed to confirm the spatial distribution of the particles. Thermogravimetric analysis was performed using a thermogravimetric analyzer (SDT500; TA instruments, New Castle, DE, USA). The samples were heated from 30°C to 800°C at a rate of 10°C min^−1^ under a nitrogen atmosphere. For fluorescence‐based colocalization analysis, EpCAM–AuMagTags (20 µL) were incubated with IFN‐γ‐treated (100 ng mL^−1^; 200 µL) SCC152 culture supernatant for 1 h at 37°C with gentle shaking followed by three washes with PBS. The samples were labeled with PE–EpCAM antibody (5 µL) and stained with PKH67 for 20 min in the dark, followed by three washes to remove excess antibody and dye. Fluorescence was observed using a confocal microscope (LSM 980; Carl Zeiss, Oberkochen, Germany) equipped with an Axio Observer inverted stand and Airyscan 2 detector. Image acquisition was performed with ZEN Blue software (version 3.9) under identical detector gain and pinhole conditions. Colocalization and Pearson's correlation coefficient were analyzed using ImageJ software (version 1.54 g; National Institutes of Health, Bethesda, MD, USA). To assess the attachment of PD‐L1^+^ EVs to the PD‐L1 substrate, EVs and EV‐bound EpCAM–AuMagTags were applied and incubated for 1 h at 37°C with gentle shaking. The surface morphology of the PD‐L1 substrate was examined using FE‐SEM.

To ensure the analytical reliability of the platform, EV capture efficiency, antibody stability, and assay reproducibility were systematically evaluated. EpCAM–AuMagTag or EpCAM–AuMag (20 µL) was incubated with EVs (100 µL, 7 × 10^8^ particles mL^−^^1^) isolated from the supernatant of IFN‐γ‐treated SCC152 (100 ng mL^−^^1^) for 1 h at 37°C. with gentle shaking. The concentration of unbound EVs remaining in the supernatant was quantified using NTA (NanoSight Pro, Malvern Panalytical). The EV capture efficiency was calculated as [(C_i_—C_s_) / C_i_] × 100, where C_i_ and C_s_ denote the initial and residual EV concentrations, respectively.

The stability of immobilized antibodies was assessed by incubating eFluor 660‐conjugated EpCAM–AuMagTag (20 µL) in plasma sample at 37°C for 0, 1, 3, and 6 h. After PBS washing, the retained fluorescence intensity was measured using a microplate reader (SpectraMax i3x). Furthermore, the assay reproducibility was validated through batch‐to‐batch (n = 3) and inter‐day (days 1, 3, and 5) experiments using IFN‐γ‐stimulated (100 ng mL^−^^1^) SCC152 supernatants. EpCAM–AuMagTag particles (20 µL) were incubated with the supernatants for 1 h at 37°C with gentle shaking. After three washes with PBS, the particles were transferred onto the PD‐L1 substrate and incubated for 1 h under the same conditions. Raman spectra were acquired at 400 points in a 20 × 20 grid with 20 µm spacing using a Raman spectrometer (LabRAM ARAMIS) equipped with a 100× objective lens (numerical aperture, 0.95; Nikon) and an Nd:YAG laser (λ = 633 nm). The laser power at the sample surface was ∼4.5 mW. The acquisition time was set to 1 s per point. The acquired spectra were processed using MATLAB (MathWorks Inc.) with Savitzky–Golay baseline correction. The characteristic Raman peaks of DTNB were analyzed to quantify PD‐L1 expression. The coefficient of variation (CV, %) was calculated as the ratio of the standard deviation (SD) to the mean, expressed as a percentage.

Finally, A549, PC‐9, FaDu, and SCC152 cell culture supernatants were collected after stimulation with IFN‐γ (0, 10, and 100 ng mL^−1^). EpCAM–AuMagTags (20 µL) were incubated with the supernatants for 1 h at 37°C with gentle shaking, followed by washing and transfer onto the PD‐L1 substrate as described above. Raman spectra were acquired and analyzed under the same conditions. To establish baseline signals, negative control (NC) samples (EpCAM–AuMagTags only, without EVs) and untreated substrate (NT) were evaluated under identical conditions. Subsequently, to validate PD‐L1–specific capture, samples were applied to an isotype antibody–coated substrate. Raman spectra were acquired and analyzed under the same conditions.

### 4.10 Human Plasma Collection

Patients with recurrent and/or metastatic NSCLC and HNSCC who received at least one dose of PD‐1/PD‐L1 inhibitors between September 2016 and July 2024 were enrolled. Peripheral blood samples were collected immediately before and after the first treatment cycle.

### 4.11 PD‐L1 Immunohistochemistry

Archived formalin‐fixed, paraffin‐embedded surgical specimens or biopsies were obtained from patients with histologically confirmed NSCLC or HNSCC. PD‐L1 expression was assessed by immunohistochemistry. The expression of PD‐L1 in tumor cells was evaluated based on the tumor cell/tumor proportion score (defined as the percentage of tumor cells with any membrane staining above background relative to that of all viable tumor cells). The cut‐off for PD‐L1 positivity was set at 1%. NSCLC and HNSCC samples were stained using the PD‐L1 IHC 22C3 PharmDx or Ventana PD‐L1 (SP142 or SP263) assay according to standard protocols. Some HNSCC samples were stained using the PD‐L1 28‐8 PharmDx assay.

### 4.12 EpCAM^+^ EV PD‐L1 Assay

To evaluate the clinical utility of the SERS‐based EpCAM^+^ EV PD‐L1 assay, 50 µL of human plasma was incubated with 20 µL of EpCAM–AuMagTags in a protein LoBind microcentrifuge tube for 1 h at 37°C with gentle shaking. Magnetic separation was used to isolate EpCAM^+^ EVs. After three washes with PBS, the complexes were transferred onto the PD‐L1 substrate in a 96‐well plate and incubated for 1 h at 37°C with gentle shaking. Following an additional washing step, the PD‐L1 substrate was mounted onto a glass slide for Raman spectroscopy. Raman spectra were measured at 400 points in a 20 × 20 grid with 20 µm spacing using a Raman spectrometer (LabRAM ARAMIS) equipped with a 100× objective lens (Nikon; numeral aperture, 0.95) and an Nd:YAG laser (λ = 633 nm). The laser power at the sample surface was ∼4.5 mW (as measured at the objective). The acquisition time was set to 1 s per point. Spectra were processed in MATLAB (MathWorks Inc.) with Savitzky–Golay baseline correction. The characteristic Raman peaks of DTNB were analyzed to determine PD‐L1 expression.

### 4.13 Single‐Cell RNA Sequencing Analysis

To evaluate the expression of *CD63*, *CD81*, and *EPCAM*, we used scRNA‐seq data derived from four *ALK*‐rearranged lung tumor samples from our previous study [61]. Following initial quality control and doublet elimination, dimensionality reduction and Uniform Manifold Approximation and Projection (UMAP) clustering were conducted with the top 2000 variable features and the first 30 principal components. To reduce potential technical variations, batch effects across the four patients were corrected using the Harmony (v1.2.4). Cluster‐specific marker genes were identified using the ‘FindAllMarkers’ function in Seurat package, and cell type identities were assigned based on reference‐based expression of cell type markers. Then, the expression of *CD63*, *CD81*, and *EPCAM* from each cell types were plotted using VlnPlot function in Seurat package.

### 4.14 Statistics

Patient demographics, clinical characteristics, and outcomes were analyzed using descriptive statistics GraphPad Prism version 8 (GraphPad Software, Boston, Massachusetts, USA). Continuous variables were compared using paired or unpaired *t*‐tests and categorical variables were compared using the Chi‐square test. Pearson's correlation analysis was used to examine the relationship between continuous variables. Treatment response was evaluated according to the Response Evaluation Criteria in Solid Tumors (RECIST) guidelines (version 1.1). The Kaplan–Meier method was used to estimate time‐to‐event outcomes. Progression‐free survival was defined as the time from treatment initiation to disease progression or death from any cause. Overall survival was defined as the time from treatment initiation to death from any cause. Durable clinical benefit was defined as a complete response, partial response or stable disease lasting longer than 6 months. Survival curves were compared using the log‐rank test. A Cox proportional hazards regression model was used to estimate the hazard ratio, and multivariate analysis was conducted to adjust variables associated with patient outcomes. Data collection and follow‐up were conducted through 31 March 2026, and all analyses were performed using this date as the data cut‐off.

### 4.15 Ethics

All procedures performed in studies involving human participants were in accordance with the ethical standards of the institutional and/or national research committee and with the 1964 Declaration of Helsinki and its later amendments or comparable ethical standards. The study was approved by the Institutional Review Board of Yonsei University College of Medicine (Seoul, Republic of Korea) (approval number 4‐2024‐0764). All participants provided written informed consent.

## Conflicts of Interest

The authors declare no conflicts of interest.

## Supporting information




**Supporting File**: advs76389‐sup‐0001‐SuppMat.docx.

## Data Availability

The data that support the findings of this study are available from the corresponding author upon reasonable request.

## References

[1] M. Oliva , A. Spreafico , M. Taberna , et al., “Immune Biomarkers of Response to Immune‐Checkpoint Inhibitors in Head and Neck Squamous Cell Carcinoma,” Annals of Oncology 30 (2019): 57–67, 10.1093/annonc/mdy507. 30462163 PMC6336003

[2] S. C. M. Lau , Y. Pan , V. Velcheti , and K. K. Wong , “Squamous Cell Lung Cancer: Current Landscape and Future Therapeutic Options,” Cancer Cell 40 (2022): 1279–1293, 10.1016/j.ccell.2022.09.018. 36270277

[3] L. Chen , “PD‐L1 and the Dawn of Modern Cancer Immunotherapy,” Nature Medicine 31 (2025): 1378, 10.1038/s41591-025-03698-4. 40325200

[4] M. Zhao , J. D. Schoenfeld , A. M. Egloff , et al., “T Cell Dynamics With Neoadjuvant Immunotherapy in Head and Neck Cancer,” Nature Reviews Clinical Oncology 22 (2025): 83–94, 10.1038/s41571-024-00969-w. 39658611

[5] Y. K. Chae , M. Othus , S. P. Patel , et al., “A Phase II Basket Trial of Dual Anti‐CTLA‐4 and Anti‐PD‐1 Blockade in Rare Tumors (DART) SWOG S1609: Durable Responses and Delayed Pseudoprogression in Small Cell Carcinoma of the Ovary, Hypercalcemic Type Cohort,” Cancer Communications 45 (2025): 976–986, 10.1002/cac2.70020. 40402690 PMC12365539

[6] S. R. Gordon , R. L. Maute , B. W. Dulken , et al., “PD‐1 Expression by Tumour‐Associated Macrophages Inhibits Phagocytosis and Tumour Immunity,” Nature 545 (2017): 495–499, 10.1038/nature22396. 28514441 PMC5931375

[7] C. Sun , R. Mezzadra , and T. N. Schumacher , “Regulation and Function of the PD‐L1 Checkpoint,” Immunity 48 (2018): 434–452, 10.1016/j.immuni.2018.03.014. 29562194 PMC7116507

[8] X. Lin , K. Kang , P. Chen , et al., “Regulatory Mechanisms of PD‐1/PD‐L1 in Cancers,” Molecular Cancer 23 (2024): 108, 10.1186/s12943-024-02023-w. 38762484 PMC11102195

[9] D. R. Camidge , R. C. Doebele , and K. M. Kerr , “Comparing and Contrasting Predictive Biomarkers for Immunotherapy and Targeted Therapy of NSCLC,” Nature Reviews Clinical Oncology 16 (2019): 341–355, 10.1038/s41571-019-0173-9. 30718843

[10] B. Burtness , K. J. Harrington , R. Greil , et al., “Pembrolizumab Alone or With Chemotherapy Versus Cetuximab With Chemotherapy for Recurrent or Metastatic Squamous Cell Carcinoma of the Head and Neck (KEYNOTE‐048): A Randomised, Open‐Label, Phase 3 Study,” The Lancet 394 (2019): 1915–1928, 10.1016/S0140-6736(19)32591-7. 31679945

[11] A. T. Ruffin , H. Li , L. Vujanovic , D. P. Zandberg , R. L. Ferris , and T. C. Bruno , “Improving Head and Neck Cancer Therapies by Immunomodulation of the Tumour Microenvironment,” Nature Reviews Cancer 23 (2023): 173–188, 10.1038/s41568-022-00531-9. 36456755 PMC9992112

[12] D. Yue , W. Wang , H. Liu , et al., “Perioperative Tislelizumab Plus Neoadjuvant Chemotherapy for Patients With Resectable Non‐Small‐Cell Lung Cancer (RATIONALE‐315): An Interim Analysis of a Randomised Clinical Trial,” The Lancet Respiratory Medicine 13 (2025): 119–129, 10.1016/S2213-2600(24)00269-8. 39581197

[13] H.‐N. Chen , K.‐H. Liang , J.‐K. Lai , et al., “EpCAM Signaling Promotes Tumor Progression and Protein Stability of PD‐L1 Through the EGFR Pathway,” Cancer Research 80 (2020): 5035–5050, 10.1158/0008-5472.CAN-20-1264. 32978170

[14] J. Park , J. S. Park , C.‐H. Huang , et al., “An Integrated Magneto‐Electrochemical Device for the Rapid Profiling of Tumour Extracellular Vesicles From Blood Plasma,” Nature Biomedical Engineering 5 (2021): 678–689, 10.1038/s41551-021-00752-7. PMC8437135 34183802

[15] Y. Liu , Y. Wang , S. Sun , et al., “Understanding the Versatile Roles and Applications of EpCAM in Cancers: From Bench to Bedside,” Experimental Hematology & Oncology 11 (2022): 97, 10.1186/s40164-022-00352-4. 36369033 PMC9650829

[16] H. Zhao , S. Pan , A. Natalia , et al., “A Hydrogel‐Based Mechanical Metamaterial for the Interferometric Profiling of Extracellular Vesicles in Patient Samples,” Nature Biomedical Engineering 7 (2023): 135–148, 10.1038/s41551-022-00954-7. 36303008

[17] Y. Lei , X. Fei , Y. Ding , et al., “Simultaneous Subset Tracing and miRNA Profiling of Tumor‐Derived Exosomes via Dual‐Surface‐Protein Orthogonal Barcoding,” Science Advances 9 (2023): adi1556, 10.1126/sciadv.adi1556. PMC10550235 37792944

[18] Y. Wu , W. Chen , J. Deng , et al., “Tumour‐Derived Microparticles Obtained Through Microwave Irradiation Induce Immunogenic Cell Death in Lung Adenocarcinoma,” Nature Nanotechnology 20 (2025): 1119–1130, 10.1038/s41565-025-01922-3. PMC12373502 40389640

[19] F. Wu , Y. Gu , B. Kang , et al., “PD‐L1 Detection on Circulating Tumor‐Derived Extracellular Vesicles (T‐EVs) From Patients With Lung Cancer,” Translational Lung Cancer Research 10, no. 6 (2021): 2441–2451, 10.21037/tlcr-20-1277. 34295653 PMC8264343

[20] R. C. Zieren , P. J. Zondervan , K. J. Pienta , A. Bex , T. M. de Reijke , and A. D. Bins , “Diagnostic Liquid Biopsy Biomarkers in Renal Cell Cancer,” Nature Reviews Urology 21 (2024): 133–157, 10.1038/s41585-023-00818-y. 37758847

[21] R. P. Carney , R. R. Mizenko , B. T. Bozkurt , et al., “Harnessing Extracellular Vesicle Heterogeneity for Diagnostic and Therapeutic Applications,” Nature Nanotechnology 20 (2025): 14–25, 10.1038/s41565-024-01774-3. PMC11781840 39468355

[22] M. Wu , G. Wang , W. Hu , Y. Yao , and X. F. Yu , “Emerging Roles and Therapeutic Value of Exosomes in Cancer Metastasis,” Molecular Cancer 18 (2019): 53, 10.1186/s12943-019-0964-8. 30925925 PMC6441156

[23] J. Zhou , Z. Wu , J. Hu , et al., “High‐Throughput Single‐EV Liquid Biopsy: Rapid, Simultaneous, and Multiplexed Detection of Nucleic Acids, Proteins, and Their Combinations,” Science Advances 6, no. 47 (2020): abc1204, 10.1126/sciadv.abc1204. PMC7679165 33219024

[24] Q.‐F. Han , W.‐J. Li , K.‐S. Hu , et al., “Exosome Biogenesis: Machinery, Regulation, and Therapeutic Implications in Cancer,” Molecular Cancer 21 (2022): 207, 10.1186/s12943-022-01671-0. 36320056 PMC9623991

[25] J. Lim , B. Kang , H. Y. Son , et al., “Microfluidic Device for One‐Step Detection of Breast Cancer‐Derived Exosomal mRNA in Blood Using Signal‐Amplifiable 3D Nanostructure,” Biosensors and Bioelectronics 197 (2022): 113753, 10.1016/j.bios.2021.113753. 34741958

[26] C.‐C. Hsu , Y. Yang , E. Kannisto , et al., “Simultaneous Detection of Tumor Derived Exosomal Protein–MicroRNA Pairs With an Exo‐PROS Biosensor for Cancer Diagnosis,” ACS Nano 17, no. 9 (2023): 8108–8122, 10.1021/acsnano.2c10970. 37129374 PMC10266547

[27] R. Kim , B. Mun , S. Lim , et al., “Colorimetric Detection of HER2‐Overexpressing‐Cancer‐Derived Exosomes in Mouse Urine Using Magnetic‐Polydiacetylene Nanoparticles,” Small 20 (2024): 2307262, 10.1002/smll.202307262. 37963850

[28] M. N. Theodoraki , S. S. Yerneni , T. K. Hoffmann , W. E. Gooding , and T. L. Whiteside , “Clinical Significance of PD‐L1^+^ Exosomes in Plasma of Head and Neck Cancer Patients,” Clinical Cancer Research 24, no. 4 (2018): 896–905, 10.1158/1078-0432.CCR-17-2664. 29233903 PMC6126905

[29] G. Chen , A. C. Huang , W. Zhang , et al., “Exosomal PD‐L1 Contributes to Immunosuppression and is Associated With anti‐PD‐1 Response,” Nature 560 (2018): 382–386, 10.1038/s41586-018-0392-8. 30089911 PMC6095740

[30] M. Poggio , T. Hu , C.‐C. Pai , et al., “Suppression of Exosomal PD‐L1 Induces Systemic Anti‐Tumor Immunity and Memory,” Cell 177 (2019): 414–427. 30951669 10.1016/j.cell.2019.02.016 PMC6499401

[31] D. Daassi , K. M. Mahoney , and G. J. Freeman , “The Importance of Exosomal PDL1 in Tumour Immune Evasion,” Nature Reviews Immunology 20 (2020): 209–215, 10.1038/s41577-019-0264-y. 31965064

[32] C. Zhang , Y. Fan , X. Che , et al., “Anti‐PD‐1 Therapy Response Predicted by the Combination of Exosomal PD‐L1 and CD28,” Frontiers in Oncology 10 (2020): 760, 10.3389/fonc.2020.00760. 32528882 PMC7266952

[33] R. Kalluri and V. S. LeBleu , “The Biology, Function, and Biomedical Applications of Exosomes,” Science 367 (2020): aau6977, 10.1126/science.aau6977. PMC7717626 32029601

[34] Z. L. Yu , J. Y. Liu , and G. Chen , “Small Extracellular Vesicle PD‐L1 in Cancer: The Knowns and Unknowns,” NPJ Precision Oncology 6 (2022): 42, 10.1038/s41698-022-00287-3. 35729210 PMC9213536

[35] C. Kilchert , S. Wittmann , and L. Vasiljeva , “The Regulation and Functions of the Nuclear RNA Exosome Complex,” Nature Reviews Molecular Cell Biology 17, no. 4 (2016): 227–239, 10.1038/nrm.2015.15. 26726035

[36] H. Shao , H. Im , C. M. Castro , X. Breakefield , R. Weissleder , and H. Lee , “New Technologies for Analysis of Extracellular Vesicles,” Chemical Reviews 118 (2018): 1917–1950, 10.1021/acs.chemrev.7b00534. 29384376 PMC6029891

[37] C. Marar , B. Starich , and D. Wirtz , “Extracellular Vesicles in Immunomodulation and Tumor Progression,” Nature Immunology 22 (2021): 560–570, 10.1038/s41590-021-00899-0. 33753940 PMC9389600

[38] X.‐H. Tang , T. Guo , X.‐Y. Gao , et al., “Exosome‐Derived Noncoding RNAs in Gastric Cancer: Functions and Clinical Applications,” Molecular Cancer 20 (2021): 99, 10.1186/s12943-021-01396-6. 34330299 PMC8323226

[39] R. Isaac , F. C. G. Reis , W. Ying , and J. M. Olefsky , “Exosomes as Mediators of Intercellular Crosstalk in Metabolism,” Cell Metabolism 33 (2021): 1744–1762, 10.1016/j.cmet.2021.08.006. 34496230 PMC8428804

[40] J. J. Lai , Z. L. Chau , S.‐Y. Chen , et al., “Exosome Processing and Characterization Approaches for Research and Technology Development,” Advanced Science 9 (2022): 2103222, 10.1002/advs.202103222. 35332686 PMC9130923

[41] H. Tang , D. Yu , J. Zhang , et al., “The New Advance of Exosome‐Based Liquid Biopsy for Cancer Diagnosis,” Journal of Nanobiotechnology 22 (2024): 610, 10.1186/s12951-024-02863-0. 39380060 PMC11463159

[42] H. L. Tran , W. Zheng , D. A. Issadore , et al., “Extracellular Vesicles for Clinical Diagnostics: From Bulk Measurements to Single‐Vesicle Analysis,” ACS Nano 19 (2025): 28021–28109, 10.1021/acsnano.5c00706. 40720603 PMC12356129

[43] D. Yu , Y. Li , M. Wang , et al., “Exosomes as a New Frontier of Cancer Liquid Biopsy,” Molecular Cancer 21 (2022): 56, 10.1186/s12943-022-01509-9. 35180868 PMC8855550

[44] C. Zhang , K. Yi , W. Zhang , et al., “Trace Analysis of Multiple Tumor Exosomal PD‐L1 Based on SERS Immunoassay Platform,” Advanced Sensor Research 2, no. 3 (2022): 2200043, 10.1002/adsr.202200043.

[45] X. Su , X. Liu , Y. Xie , et al., “Integrated SERS‐Vertical Flow Biosensor Enabling Multiplexed Quantitative Profiling of Serological Exosomal Proteins in Patients for Accurate Breast Cancer Subtyping,” ACS Nano 17 (2023): 4077–4088, 10.1021/acsnano.3c00449. 36758150

[46] W. Ge , Z. Mu , S. Yang , et al., “Biosensor‐Based Methods for Exosome Detection With Applications to Disease Diagnosis,” Biosensors and Bioelectronics 279 (2025): 117362, 10.1016/j.bios.2025.117362. 40157151

[47] L. K. Chin , T. Son , J.‐S. Hong , et al., “Plasmonic Sensors for Extracellular Vesicle Analysis: From Scientific Development to Translational Research,” ACS Nano 14 (2020): 14528–14548, 10.1021/acsnano.0c07581. 33119256 PMC8423498

[48] S. Laing , S. Sloan‐Dennison , K. Faulds , and D. Graham , “Surface Enhanced Raman Scattering for Biomolecular Sensing in Human Healthcare Monitoring,” ACS Nano 19 (2025): 8381–8400, 10.1021/acsnano.4c15877. 40014676 PMC11912579

[49] X. Bi , D. M. Czajkowsky , Z. Shao , and J. Ye , “Digital Colloid‐Enhanced Raman Spectroscopy by Single‐Molecule Counting,” Nature 628 (2024): 771–775, 10.1038/s41586-024-07218-1. 38632399

[50] J. Li , A. Wuethrich , A. A. I. Sina , et al., “A Digital Single‐Molecule Nanopillar SERS Platform for Predicting and Monitoring Immune Toxicities in Immunotherapy,” Nature Communications 12 (2021): 1087, 10.1038/s41467-021-21431-w. PMC7889912 33597530

[51] Q. Zhao , H. Hilal , J. Kim , et al., “All‐Hot‐Spot Bulk Surface‐Enhanced Raman Scattering (SERS) Substrates: Attomolar Detection of Adsorbates With Designer Plasmonic Nanoparticles,” Journal of the American Chemical Society 144 (2022): 13285–13293, 10.1021/jacs.2c04514. 35839479

[52] D. H. Kim , H. Kim , Y. J. Choi , et al., “Exosomal PD‐L1 Promotes Tumor Growth Through Immune Escape in Non‐Small Cell Lung Cancer,” Experimental & Molecular Medicine 51 (2019): 1–13, 10.1038/s12276-019-0295-2. PMC6802663 31399559

[53] F. Xie , M. Xu , J. Lu , L. Mao , and S. Wang , “The Role of Exosomal PD‐L1 in Tumor Progression and Immunotherapy,” Molecular Cancer 18 (2019): 146, 10.1186/s12943-019-1074-3. 31647023 PMC6813045

[54] B. Kang , S. Han , H. Y. Son , et al., “Immunomagnetic Microfluidic Integrated System for Potency‐Based Multiple Separation of Heterogeneous Stem Cells With High Throughput Capabilities,” Biosensors and Bioelectronics 194 (2021): 113576, 10.1016/j.bios.2021.113576. 34454345

[55] B. Mun , R. Kim , H. Jeong , et al., “An Immuno‐Magnetophoresis‐Based Microfluidic Chip to Isolate and Detect HER2‐Positive Cancer‐Derived Exosomes via Multiple Separation,” Biosensors and Bioelectronics 239 (2023): 115592, 10.1016/j.bios.2023.115592. 37603987

[56] R. Kim , B. Mun , J. Lim , et al., “Portable Monitoring System for Assessing Therapeutic Efficacy in HER2‐Overexpressing Breast Cancer: One‐Step Simultaneous Detection of Cancer‐Derived Exosomal Proteins and mRNA in Urine,” Advanced Functional Materials 34 (2024): 2410817, 10.1002/adfm.202410817.

[57] Y. Xue , X. Li , H. Li , and W. Zhang , “Quantifying Thiol–Gold Interactions Towards the Efficient Strength Control,” Nature Communications 5 (2014): 4348, 10.1038/ncomms5348. 25000336

[58] J. Kim , H. Y. Son , S. Lee , et al., “Deep Learning‐Assisted Monitoring of Trastuzumab Efficacy in HER2‐Overexpressing Breast Cancer via SERS Immunoassays of Tumor‐Derived Urinary Exosomal Biomarkers,” Biosensors and Bioelectronics 258 (2024): 116347, 10.1016/j.bios.2024.116347. 38723332

[59] B. Mun , H. Jeong , R. Kim , et al., “3D‐Nanostructured Microfluidic Device Arranged in a Herringbone Pattern for the Highly Effective Capture of HER2‐Positive Cancer‐Derived Exosomes in Urine,” Chemical Engineering Journal 482 (2024): 148851, 10.1016/j.cej.2024.148851.

[60] Z. Yin , M. Yu , T. Ma , et al., “Mechanisms Underlying Low‐Clinical Responses to PD‐1/PD‐L1 Blocking Antibodies in Immunotherapy of Cancer: A Key Role of Exosomal PD‐L1,” Journal for ImmunoTherapy of Cancer 9 (2021): 001698, 10.1136/jitc-2020-001698. PMC7818841 33472857

[61] S. Baek , E. Sung , G. Kim , et al., “Single‐Cell Multi‐Omics Reveals Tumor Microenvironment Factors Underlying Poor Immunotherapy Responses in ALK‐Positive Lung Cancer,” Cancer Communications 45 (2025): 422–427, 10.1002/cac2.12658. 39754710 PMC11999889

