# Supplementary material for: EpCAM+ Extracellular Vesicle PD‐L1 Dynamics as a Predictive Biomarker of Immune Checkpoint Blockade Response
Source: Adv Sci (Weinh). 2026 Jul 3:e76389. Online ahead of print. doi: 10.1002/advs.76389 (PMC13334590; doi:10.1002/advs.76389)
Supplement: Supplementary file 1 — Supporting File: advs76389‐sup‐0001‐SuppMat.docx. [file ADVS-9999-e76389-s001.docx]

Supporting Information

EpCAM⁺ Extracellular Vesicle PD-L1 Dynamics as a Predictive Biomarker of Immune Checkpoint Blockade Response

Byeonggeol Mun^1^, Jinyoung Kim^2^, Chang Gon Kim^3^, Seokhyeong Go^3^, Mina Han^3^, Yujin Ouck^1^, Soojin Jang^2,4^, Seong Uk Son^2,4^, Gamin Kim^3^, Wonrak Son^3^, Eunjung Kim^5^, Min Hee Hong^3^, Ja Hyun Yeo^3^, Eun-Kyung Lim^2,4,6^*, Hye Ryun Kim^3^* & Seungjoo Haam^1^*

B. Mun, Y. Ouck, S. Haam
Department of Chemical and Biomolecular Engineering, College of Engineering, Yonsei University, Seoul, Republic of Korea
E-mail: haam@yonsei.ac.kr (S. Haam)

J. Kim, S. Jang, S. U. Son, E.-K. Lim
Bionanotechnology Research Center, Korea Research Institute of Bioscience and Biotechnology (KRIBB), Daejeon, Republic of Korea
E-mail: eklim1112@kribb.re.kr (E.-K. Lim)

C. G. Kim, S. Go, M. Han, G. Kim, W. Son, M. H. Hong, J. H. Yeo, H. R. Kim
Division of Medical Oncology, Department of Internal Medicine, Yonsei University College of Medicine, Seoul, Republic of Korea
E-mail: nobelg@yuhs.ac (H. R. Kim)

S. Jang, S. U. Son, E.-K. Lim
Department of Nanobiotechnology, KRIBB School of Biotechnology, University of Science and Technology, Daejeon, Republic of Korea

E. Kim
Department of Bioengineering and Nano-Bioengineering, Research Center for Bio Materials and Process Development, Incheon National University, Incheon, Republic of Korea

E.-K. Lim
School of Pharmacy, Sungkyunkwan University, Suwon, Republic of Korea.


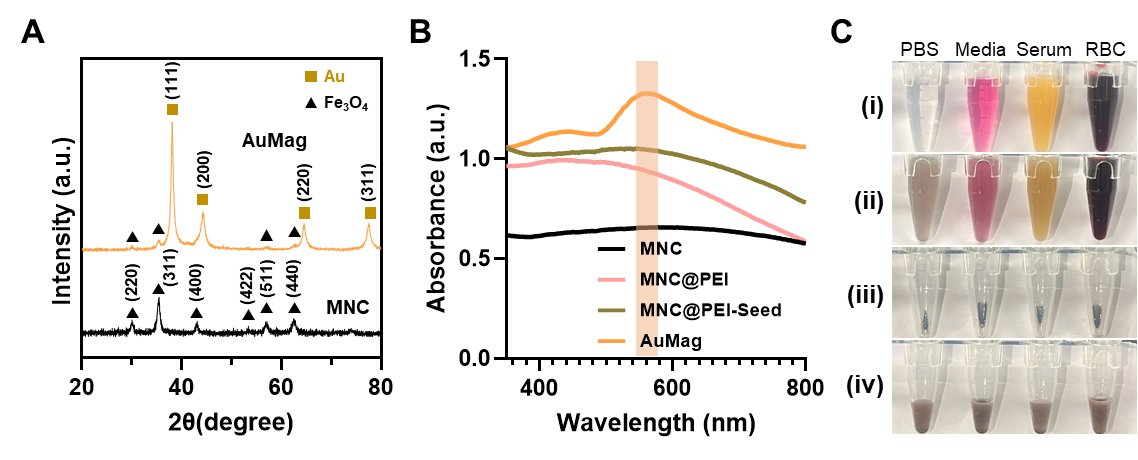


**Figure S1. Characterization of AuMag.** (A) X-ray diffraction (XRD) patterns of MNCs and AuMags. (B) UV-Vis absorbance spectra of MNCs at different synthesis stages, including bare, PEI-coated, Au seed-functionalized, and Au-coated forms. (C) Sequential photographic images illustrating the magnetic capture process of AuMags in various solutions (PBS, Media, Serum, and RBC solution), showing (i) the initial solution, (ii) the dispersion of AuMags, (iii) the magnetically captured AuMags, and (iv) redispersion in pure water.


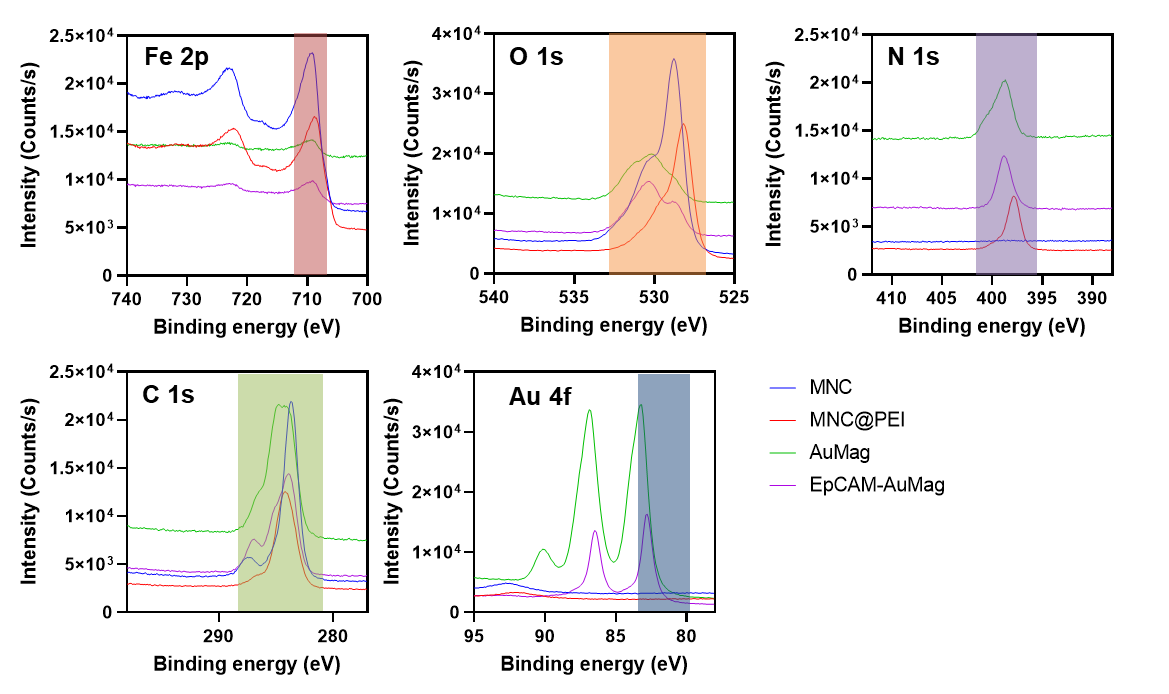


**Figure S2.** X-ray photoelectron spectroscopy (XPS) spectra of Fe 2p, O 1s, N 1s, C 1s, and Au 4f at different synthesis stages, including bare, PEI-coated, Au-coated, and antibody-functionalized forms.


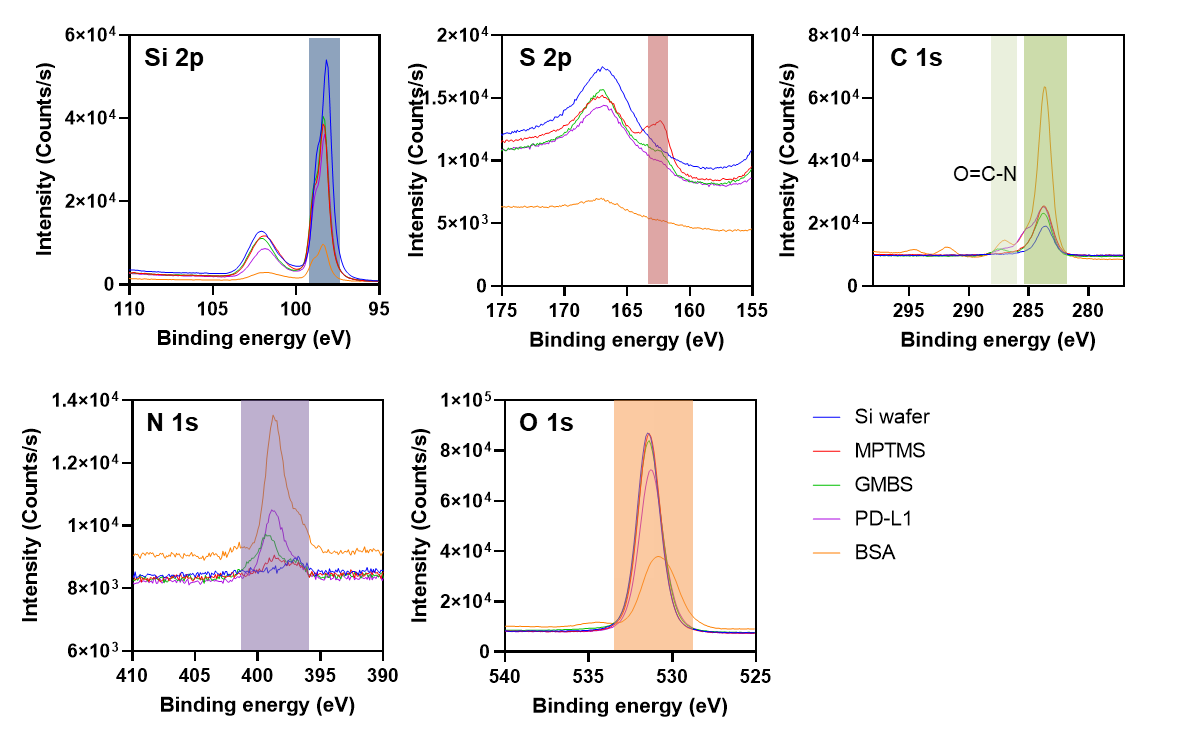


**Figure S3.** X-ray photoelectron spectroscopy (XPS) spectra of Si 2p, S 2p, C 1s, N 1s, and O 1s at different surface modifications stages, including bare Si wafer, sulfhydryl functionalized surface, Amin-to-sulfhydryl cross linker modification, antibody attachment, and antifouling coating.


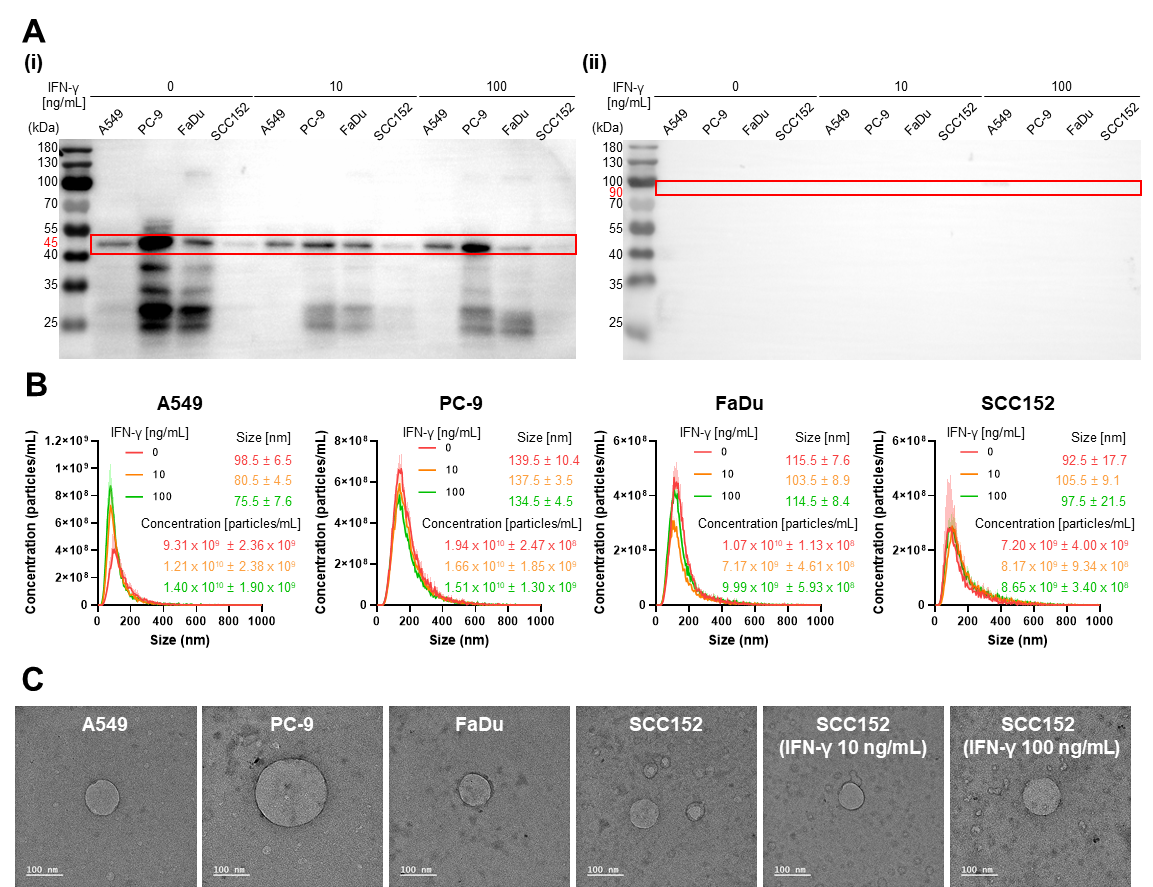


**Figure S4. Characterization of extracellular vesicles (EVs) derived from NSCLC and HNSCC cell lines.** (A) Western blot analysis of the EV-associated marker TSG101 (~45 kDa) (i) and the intracellular negative marker calnexin (~90 kDa) (ii). (B) Nanoparticle tracking analysis (NTA) showing the size distribution and concentration of isolated EVs. (C) Representative transmission electron microscopy (TEM) images of isolated EVs.


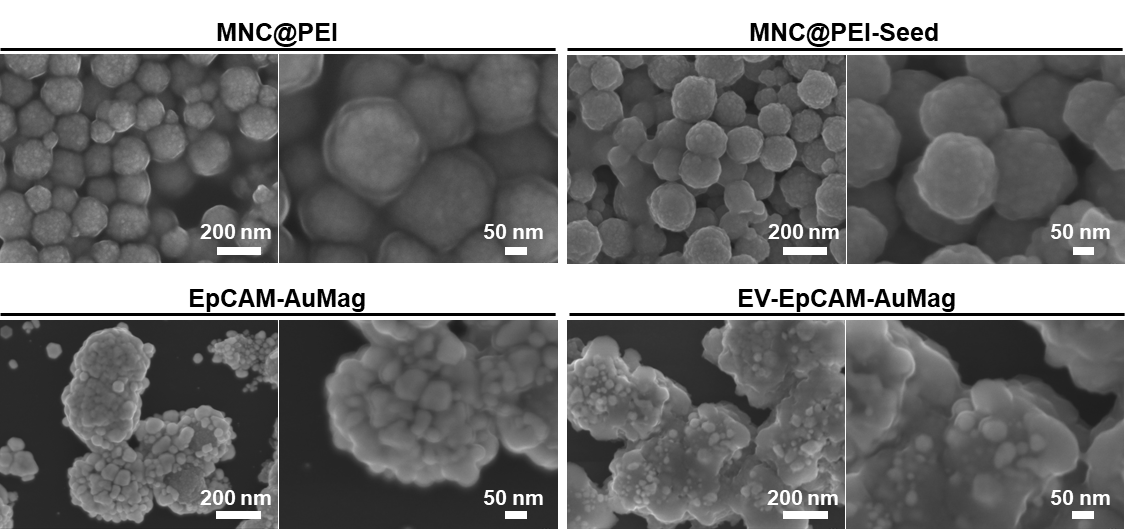


**Figure S5.** Field-emission scanning electron microscope (FE-SEM) images of PEI-coated MNCs, Au seed-functionalized MNCs, antibody-functionalized AuMags and EpCAM^+^ EVs bound EpCAM-AuMags.


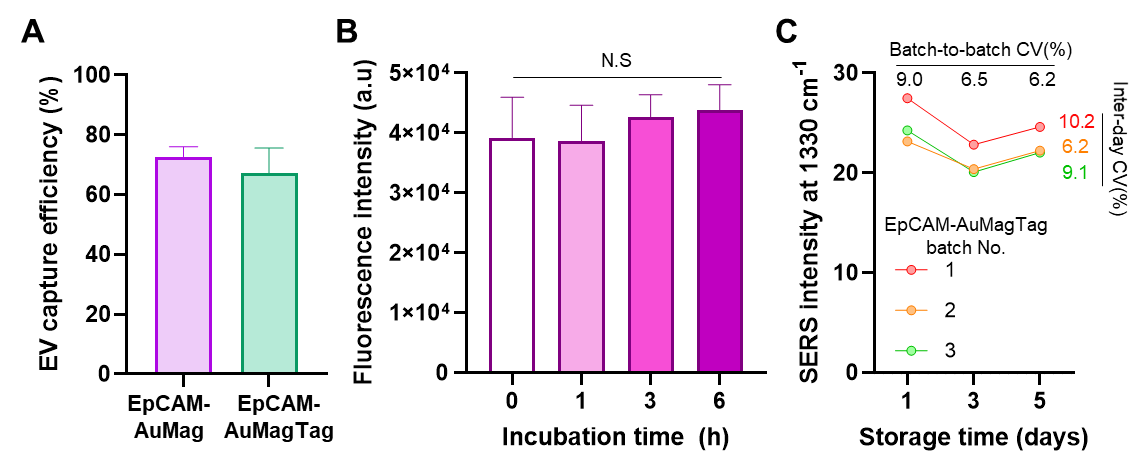


**Figure S6. Evaluation of EV capture performance, antibody stability, and assay reproducibility of EpCAM–AuMagTag.** (A) Comparison of EV isolation efficiency between EpCAM–AuMag and EpCAM–AuMagTag. (B) Stability of EpCAM antibodies on EpCAM–AuMagTag particles in plasma, assessed by fluorescence intensity over time (0, 1, 3, and 6 h). Data represent mean ± SD (n = 3). Significance levels are denoted as N.S (not significant, p > 0.05). (C) Reproducibility of the SERS platform evaluated by batch-to-batch and inter-day variations, with results expressed as the coefficients of variation (CV, %).


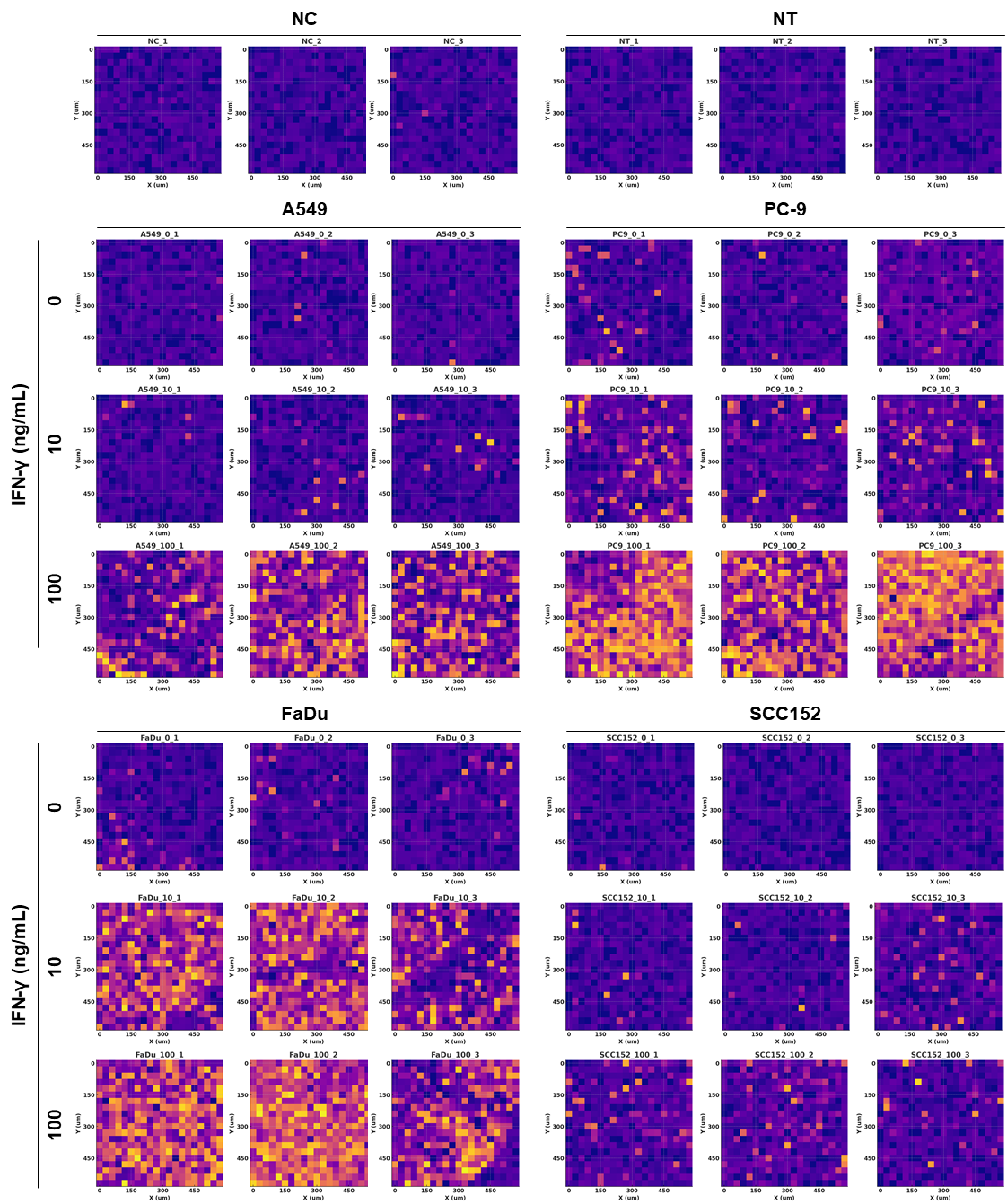


**Figure S7.** Heatmap of the characteristic SERS peak at 1330 cm-1, quantified from the Raman spectra obtained from conditioned media of A549, PC-9, FaDu, and SCC152 cell lines treated with increasing concentrations of IFN-γ (0, 10, and 100 ng/mL), as well as from negative controls without media (NC) and PD-L1 substrates without treatment (NT), for each replicates (n=3).


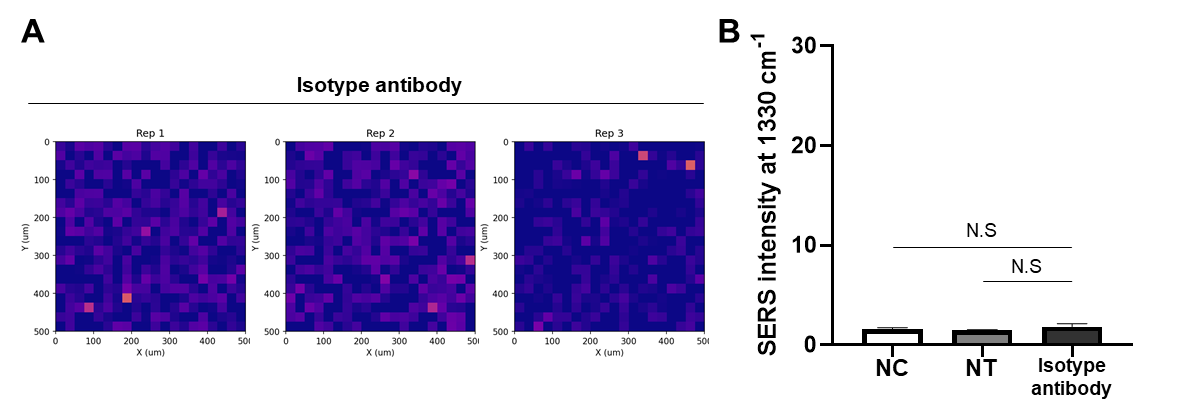


**Figure S8. Validation of non–specific capture using an isotype antibody control.** (A) Heatmap of the characteristic SERS peak at 1330 cm^-1^, quantified from the Raman spectra of the isotype antibody-coated substrate using conditioned media of SCC152 cells treated with IFN-γ (100 ng/mL). (B) Quantitative comparison of SERS intensities for the isotype control, negative control (NC; EpCAM–AuMagTags only), and untreated PD-L1 substrate (NT). Significance levels are denoted as N.S (not significant, p > 0.05).


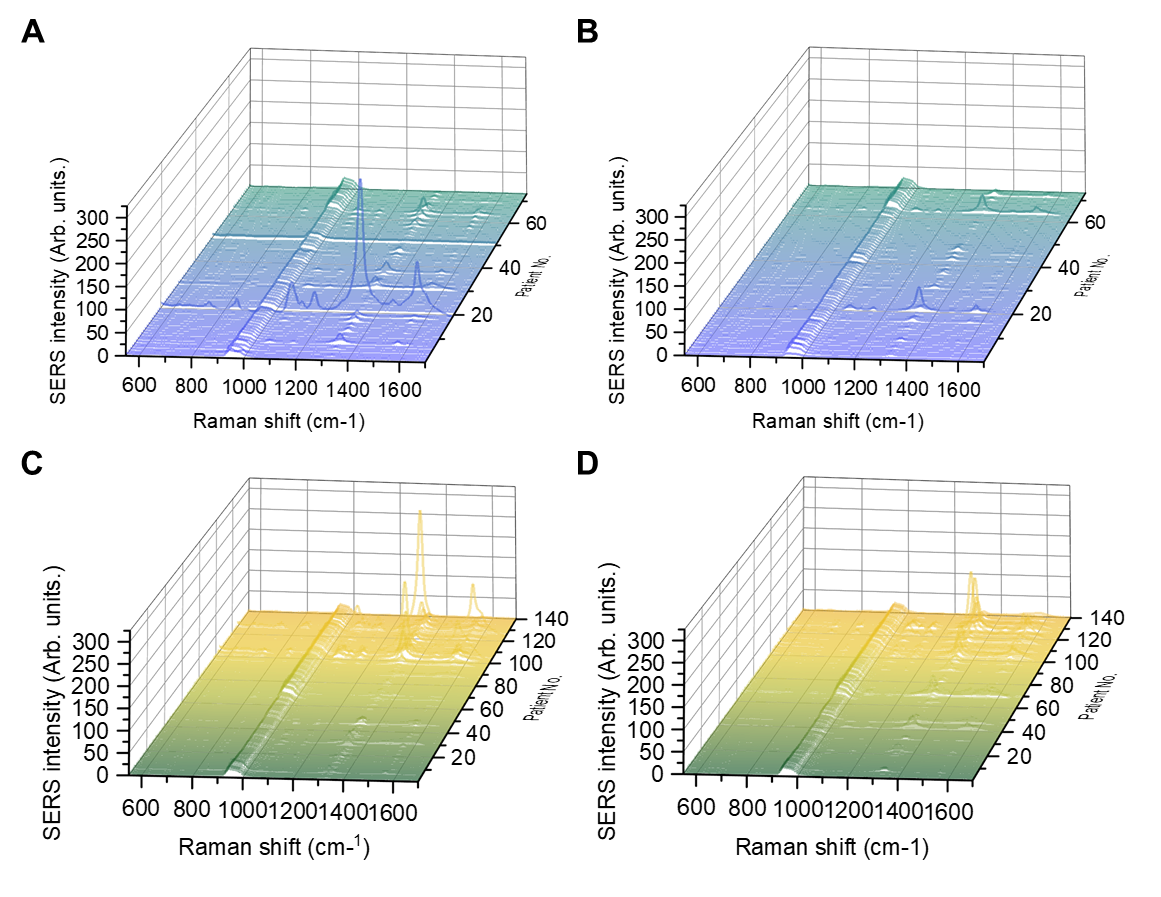


**Figure S9. Raman spectra of plasma from HNSCC and NSCLC patients treated with PD-(L)1 inhibitors, analyzed using the EpCAM^+^ EV PD-L1 assay.** Spectra from (A) Pre-treatment and (B) post-treatment HNSCC patients (n = 73). Spectra from (C) Pre-treatment and (D) post-treatment NSCLC patients (n = 140)


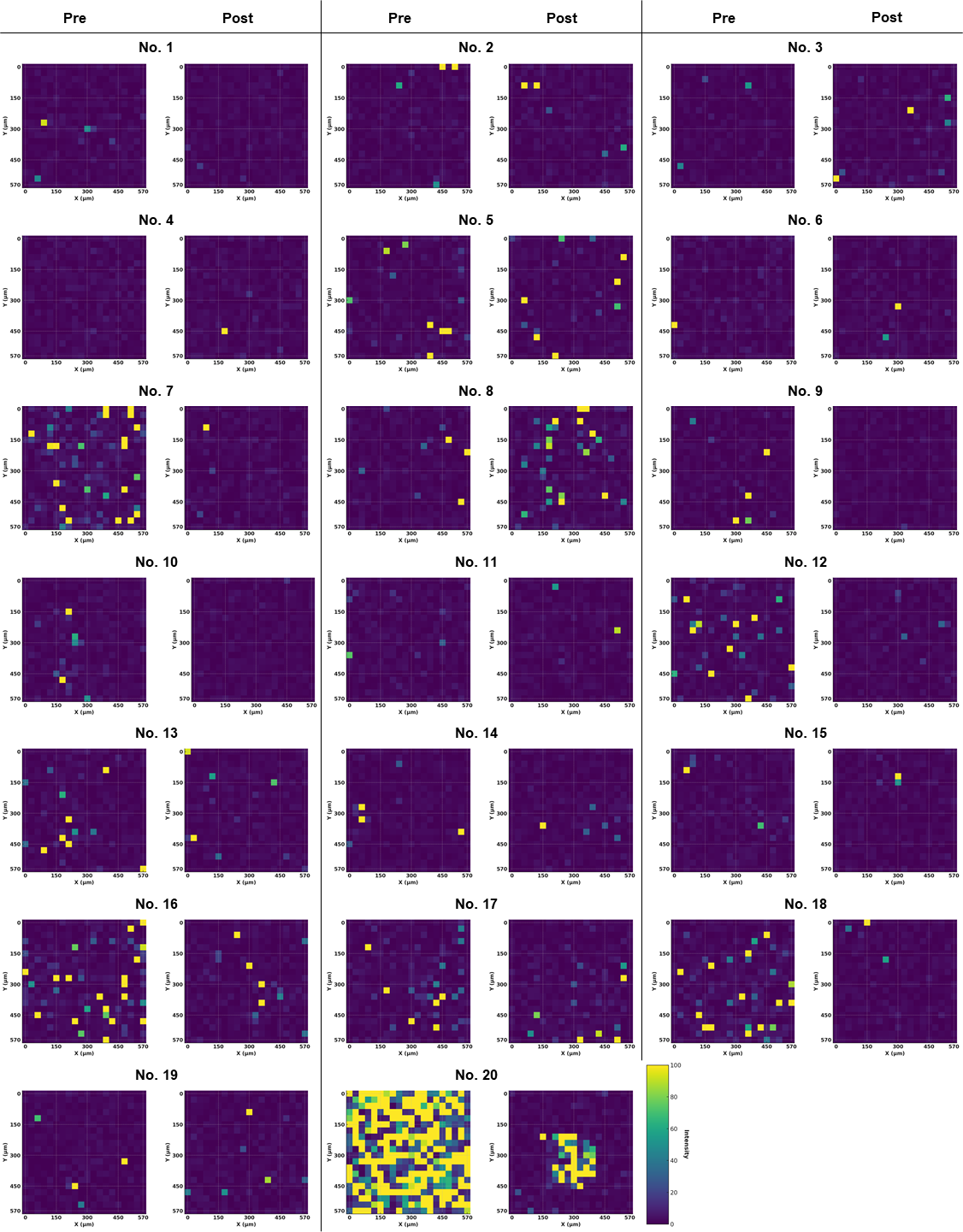


**Figure S10.** Heatmap of the characteristic SERS peak at 1330 cm^-1^, quantified from the Raman spectra of 73 HNSCC patients treated with PD-(L)1 inhibitors using the EpCAM^+^ EV PD-L1 assay. Data is shown separately for pre-treatment and post-reatment samples with 20 patients (No.1-20).


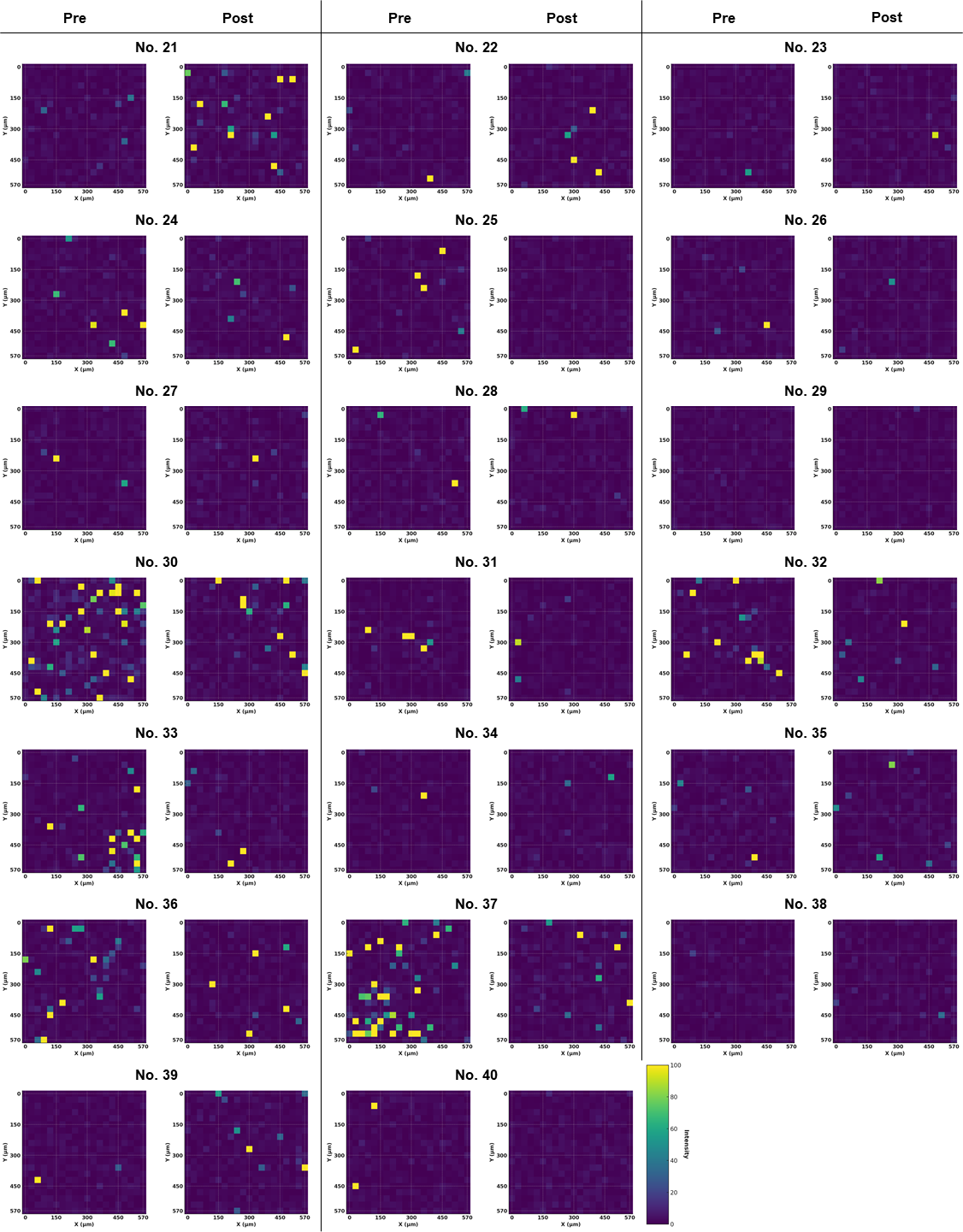


**Figure S11.** Heatmap of the characteristic SERS peak at 1330 cm^-1^, quantified from the Raman spectra of 73 HNSCC patients treated with PD-(L)1 inhibitors using the EpCAM^+^ EV PD-L1 assay. Data is shown separately for pre-treatment and post-reatment samples with 20 patients (No.21-40).


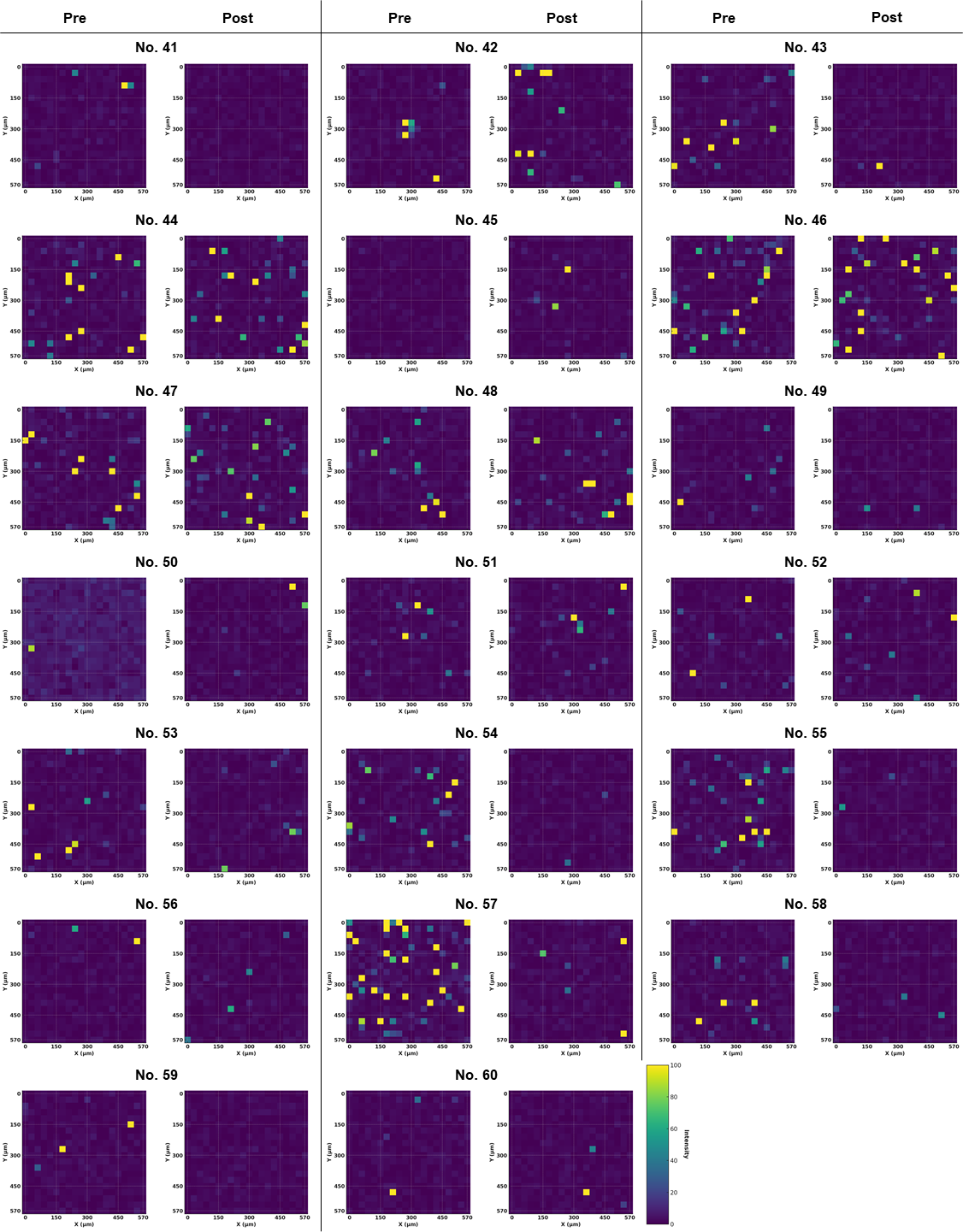


**Figure S12.** Heatmap of the characteristic SERS peak at 1330 cm^-1^, quantified from the Raman spectra of 73 HNSCC patients treated with PD-(L)1 inhibitors using the EpCAM^+^ EV PD-L1 assay. Data is shown separately for pre-treatment and post-reatment samples with 20 patients (No.41-60).


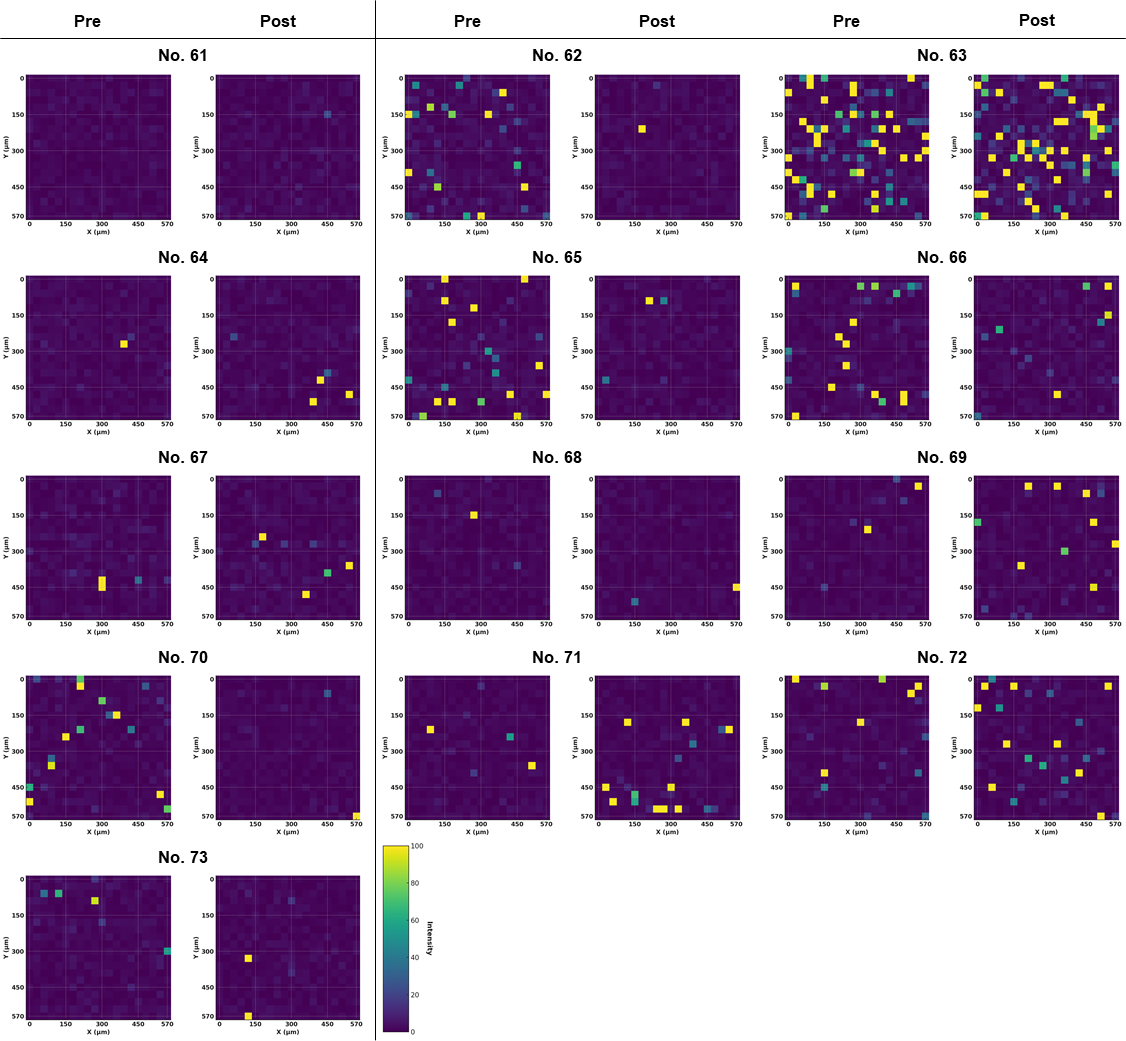


**Figure S13.** Heatmap of the characteristic SERS peak at 1330 cm^-1^, quantified from the Raman spectra of 73 HNSCC patients treated with PD-(L)1 inhibitors using the EpCAM^+^ EV PD-L1 assay. Data is shown separately for pre-treatment and post-treatment samples with 13 patients (No.61-73).


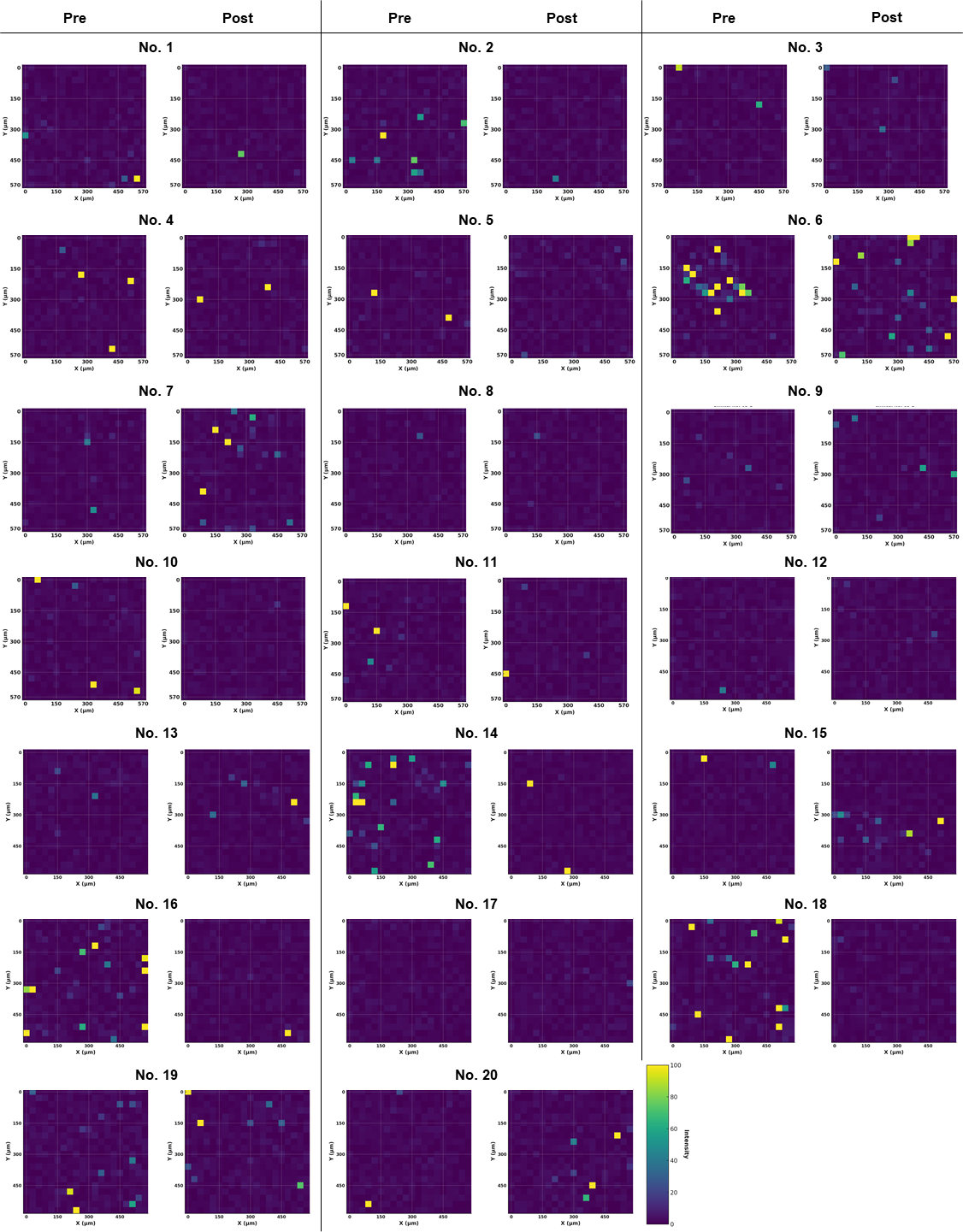


**Figure S14.** Heatmap of the characteristic SERS peak at 1330 cm^-1^, quantified from the Raman spectra of 140 NSCLC patients treated with PD-(L)1 inhibitors using the EpCAM^+^ EV PD-L1 assay. Data are shown separately for pre-treatment and post-treatment samples with 20 patients (No.1-20).


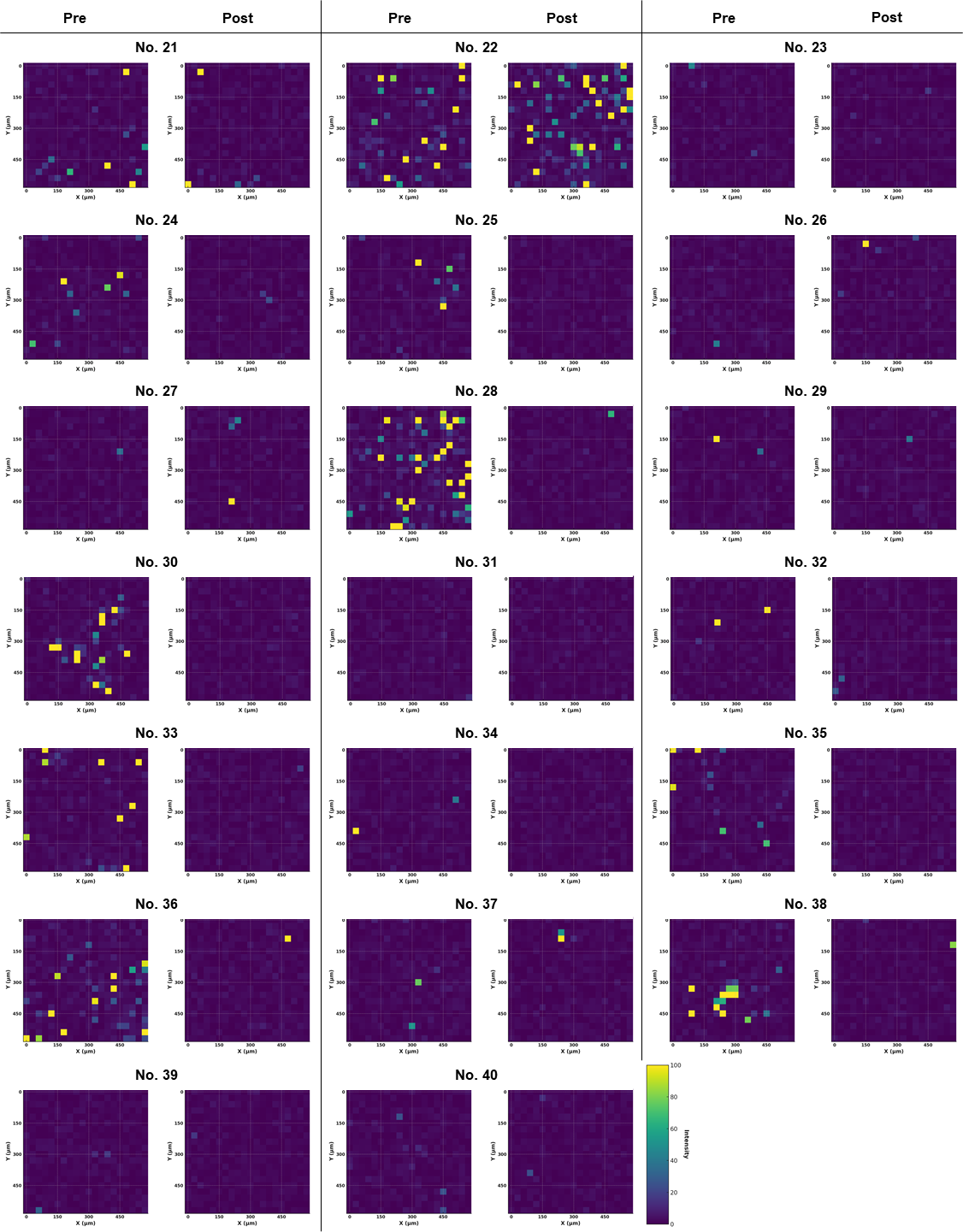


**Figure S15.** Heatmap of the characteristic SERS peak at 1330 cm^-1^, quantified from the Raman spectra of 140 NSCLC patients treated with PD-(L)1 inhibitors using the EpCAM^+^ EV PD-L1 assay. Data are shown separately for pre-treatment and post-treatment samples with 20 patients (No.21-40).


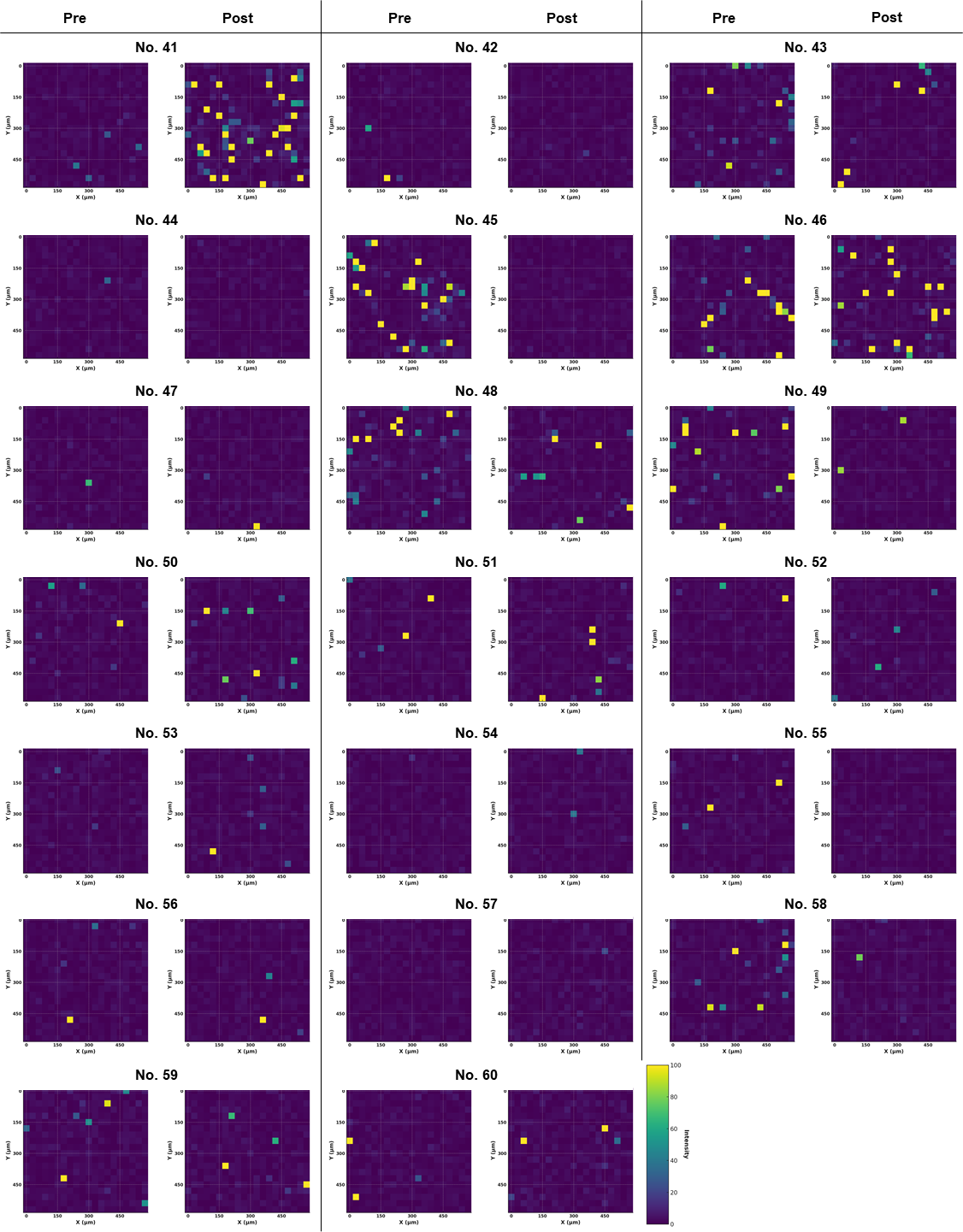


**Figure S16.** Heatmap of the characteristic SERS peak at 1330 cm^-1^, quantified from the Raman spectra of 140 NSCLC patients treated with PD-(L)1 inhibitors using the EpCAM^+^ EV PD-L1 assay. Data are shown separately for pre-treatment and post-treatment samples with 20 patients (No.41-60).


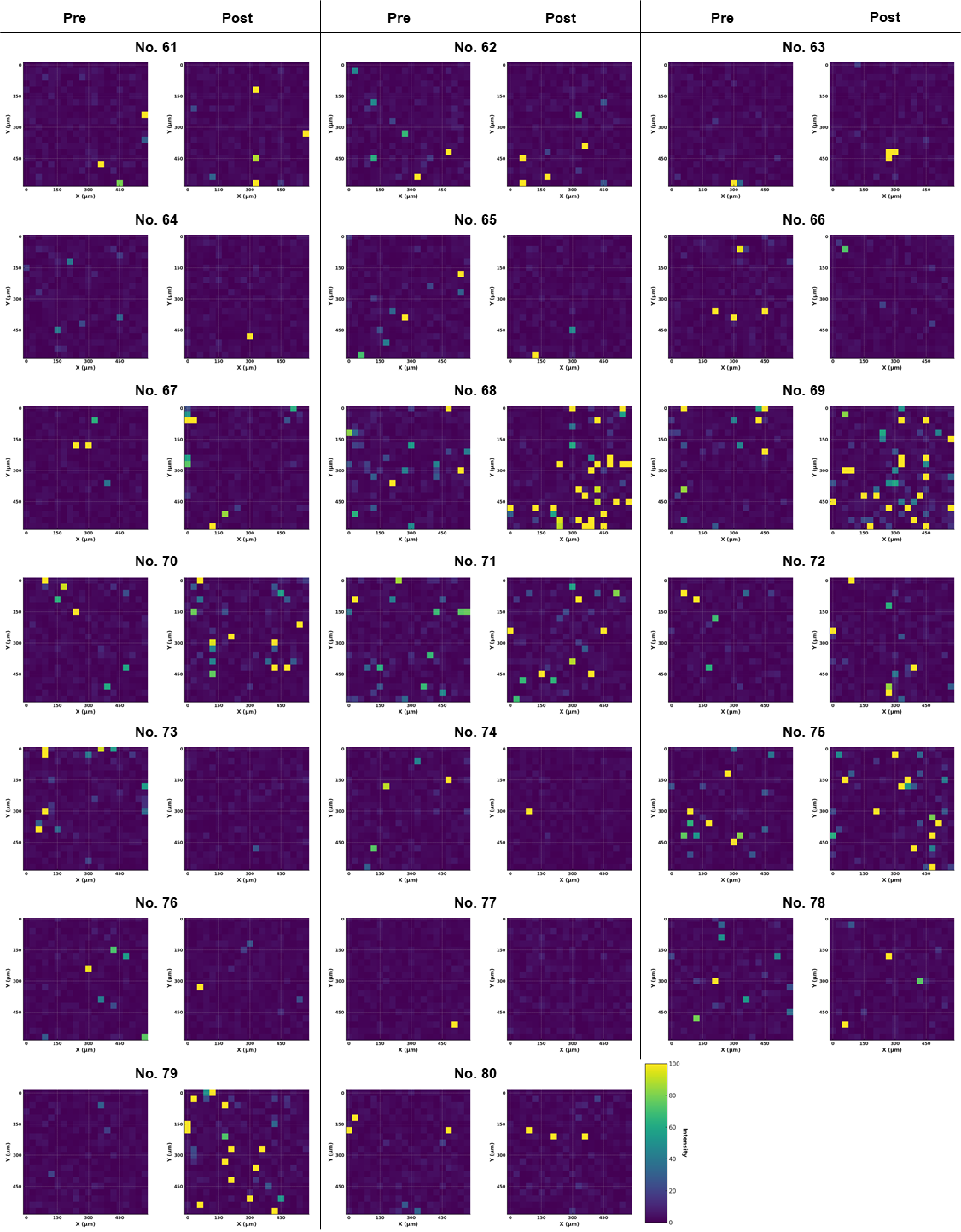


**Figure S17.** Heatmap of the characteristic SERS peak at 1330 cm^-1^, quantified from the Raman spectra of 140 NSCLC patients treated with PD-(L)1 inhibitors using the EpCAM^+^ EV PD-L1 assay. Data are shown separately for pre-treatment and post-treatment samples with 20 patients (No.61-80).


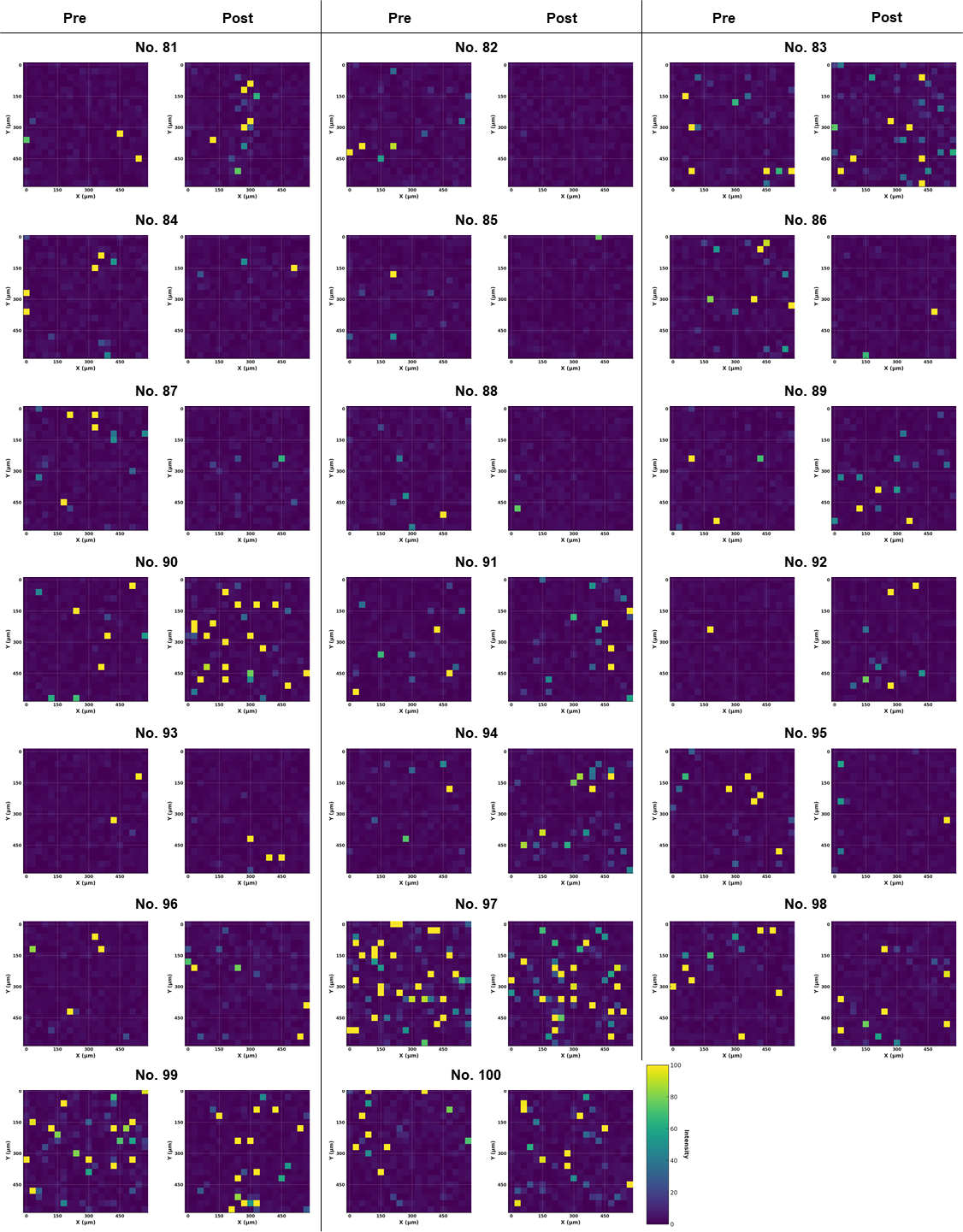


**Figure S18.** Heatmap of the characteristic SERS peak at 1330 cm^-1^, quantified from the Raman spectra of 140 NSCLC patients treated with PD-(L)1 inhibitors using the EpCAM^+^ EV PD-L1 assay. Data are shown separately for pre-treatment and post-treatment samples with 20 patients (No.81-100).


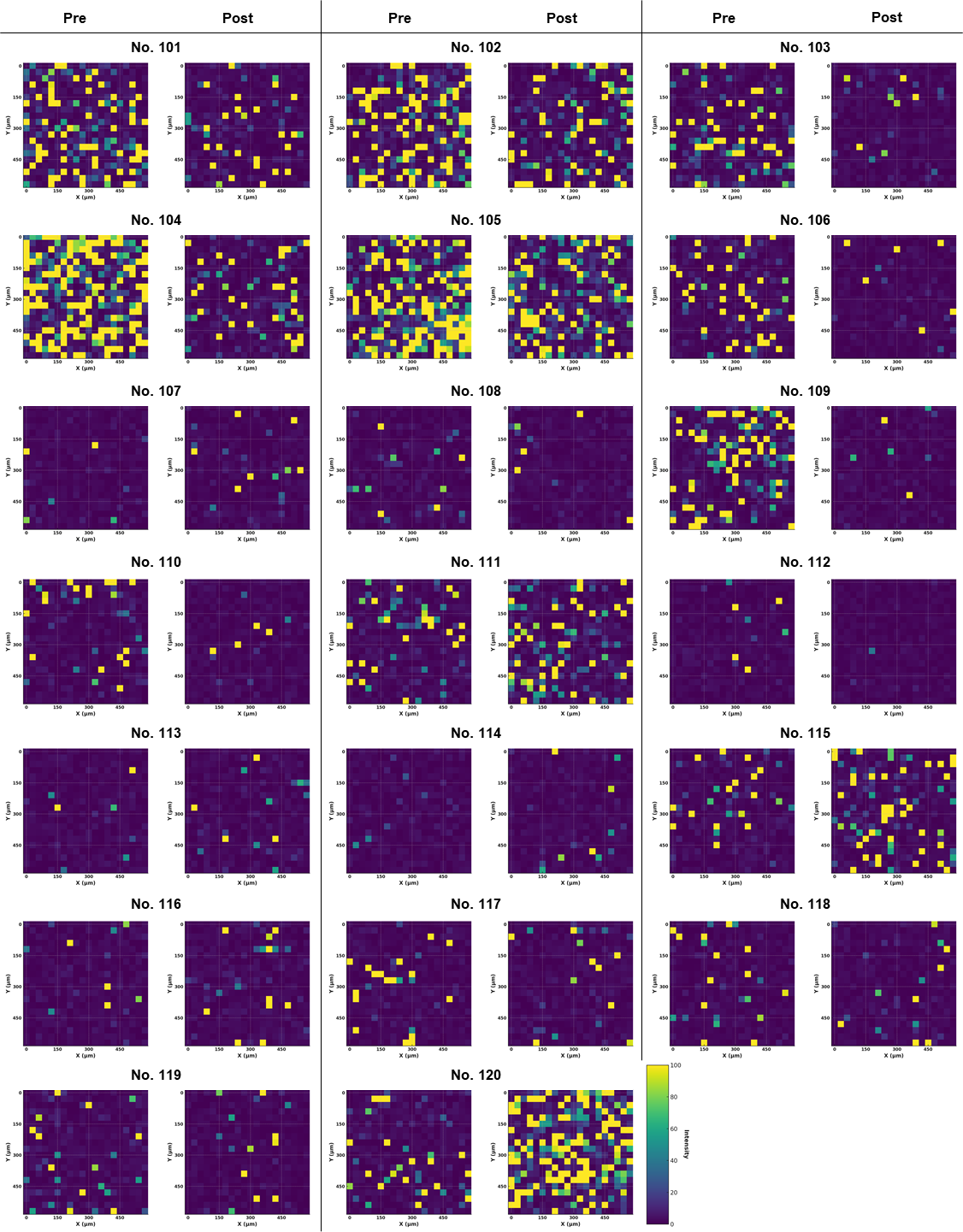


**Figure S19.** Heatmap of the characteristic SERS peak at 1330 cm^-1^, quantified from the Raman spectra of 140 NSCLC patients treated with PD-(L)1 inhibitors using the EpCAM^+^ EV PD-L1 assay. Data are shown separately for pre-treatment and post-treatment samples with 20 patients (No.101-120).


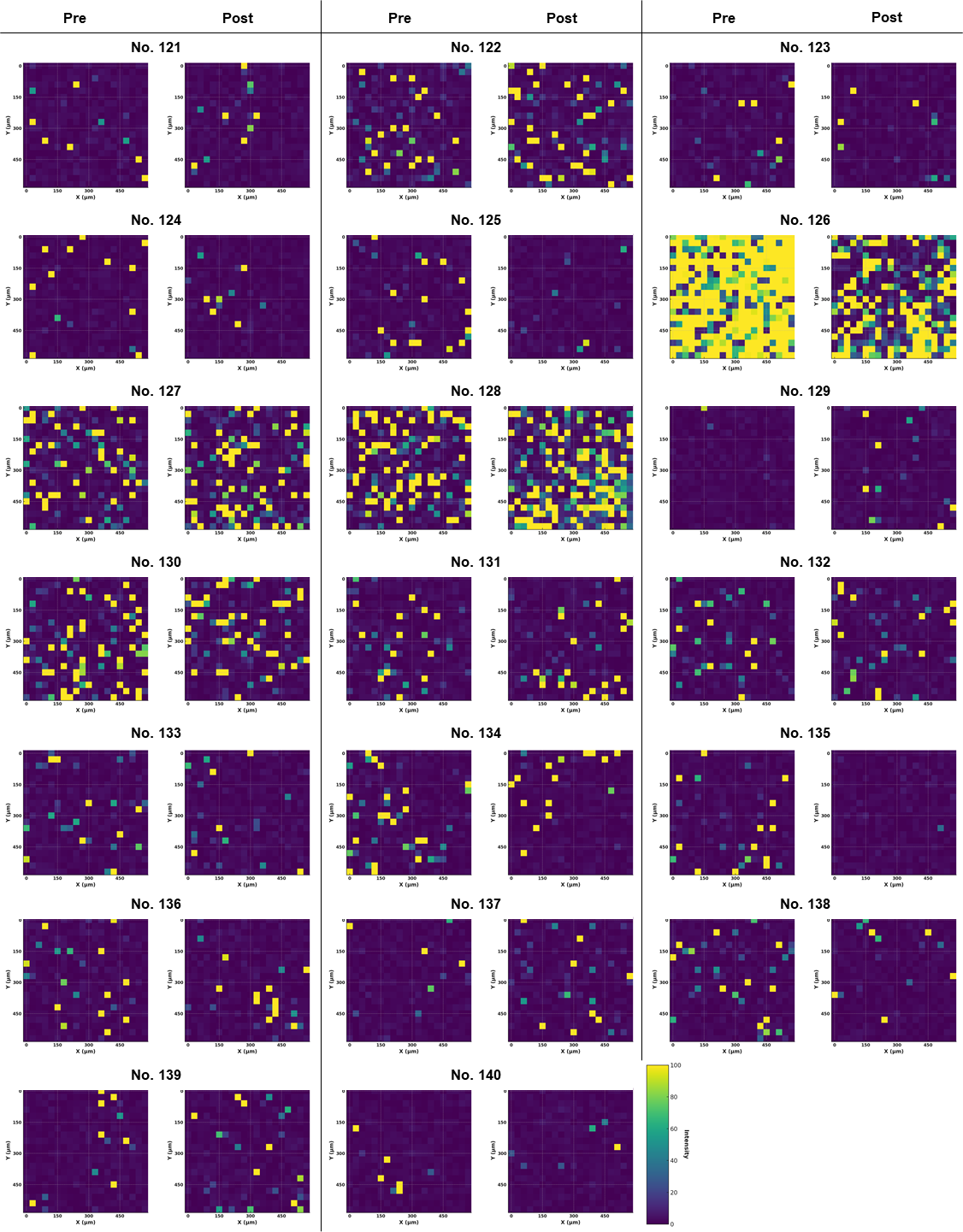


**Figure S20.** Heatmap of the characteristic SERS peak at 1330 cm^-1^, quantified from the Raman spectra of 140 NSCLC patients treated with PD-(L)1 inhibitors using the EpCAM^+^ EV PD-L1 assay. Data are shown separately for pre-treatment and post-treatment samples with 20 patients (No.121-140).


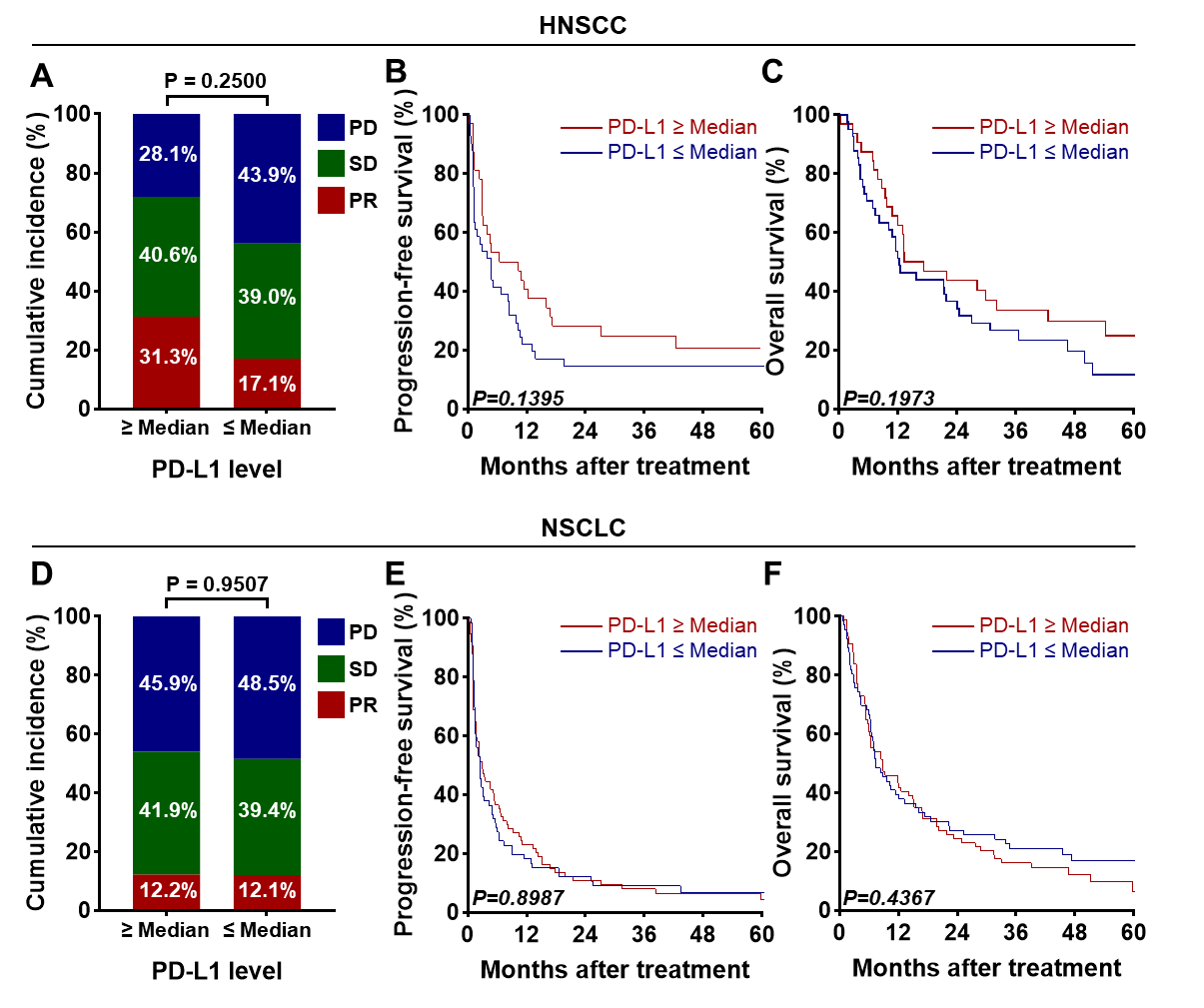


**Figure S21. Basline EpCAM^+^** **EV PD-L1 and tratment outcomes.** (A) Best response to PD-(L)1 inhibitors according to baseline EpCAM^+^ EV PD-L1 expression in patients with HNSCC. (B) Progression-free survival after treatment with PD-(L)1 inhibitors according to baseline in EpCAM^+^ EV PD-L1 expression in patients with HNSCC. (C) Overall survival after treatment with PD-(L)1 inhibitors according to baseline in EpCAM^+^ EV PD-L1 expression in patients with HNSCC. (D) Best response to PD-(L)1 inhibitors according to baseline in EpCAM^+^ EV PD-L1 expression in patients with NSCLC. (E) Progression-free survival after treatment with PD-(L)1 inhibitors according to baseline in EpCAM^+^ EV PD-L1 expression in patients with NSCLC. (F) Overall survival after treatment with PD-(L)1 inhibitors according to baseline in EpCAM^+^ EV PD-L1 expression in patients with NSCLC.


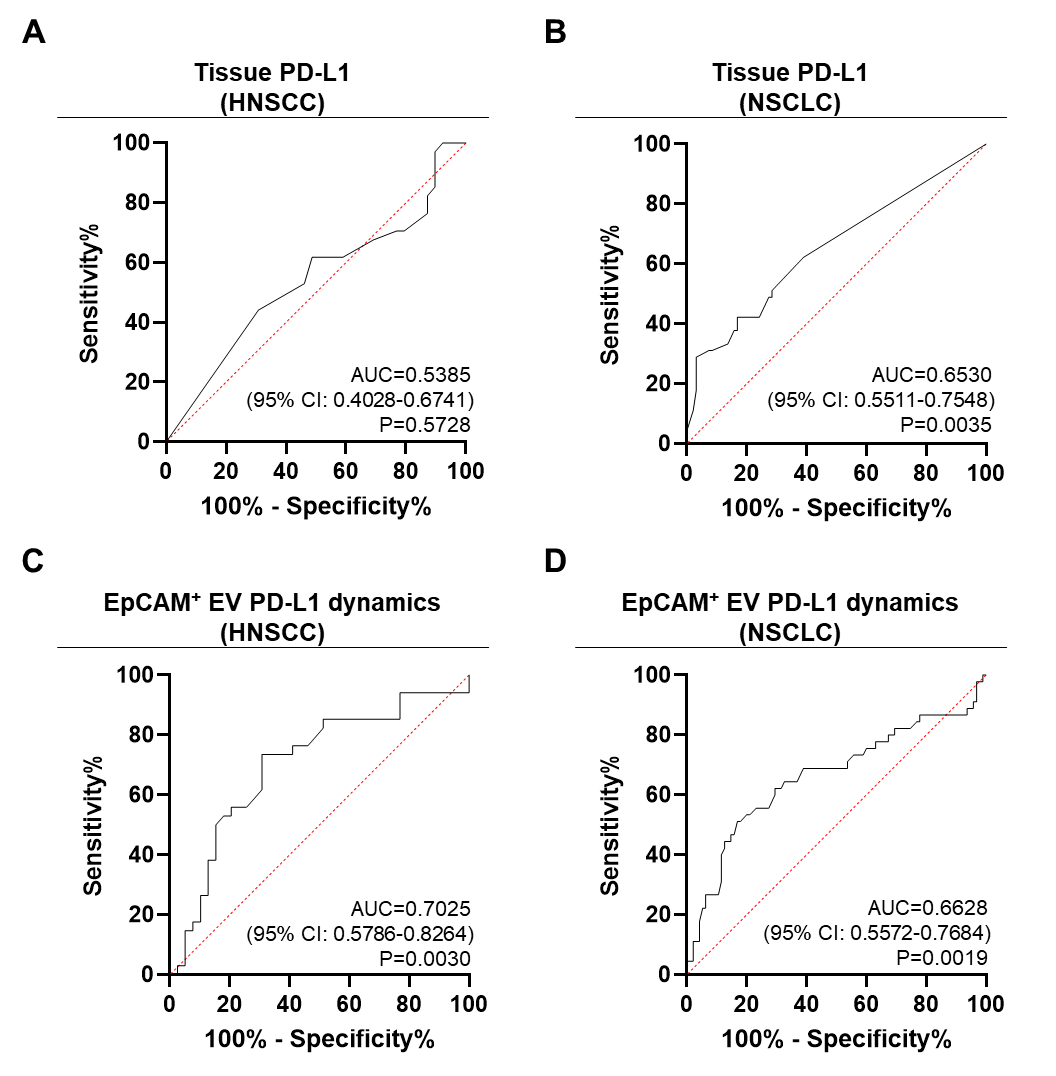


**Figure S22. Receiver operating characteristic (ROC) curve analysis for durable clinical benefit** (defined as a complete response, partial response or stable disease lasting longer than 6 months). ROC curves for tumor PD-L1 expression during PD-(L)1 inhibitor treatment in (A) HNSCC and (B) NSCLC. ROC curves for dynamic changes in EpCAM⁺ EV PD-L1 levels in (C) HNSCC and (D) NSCLC.


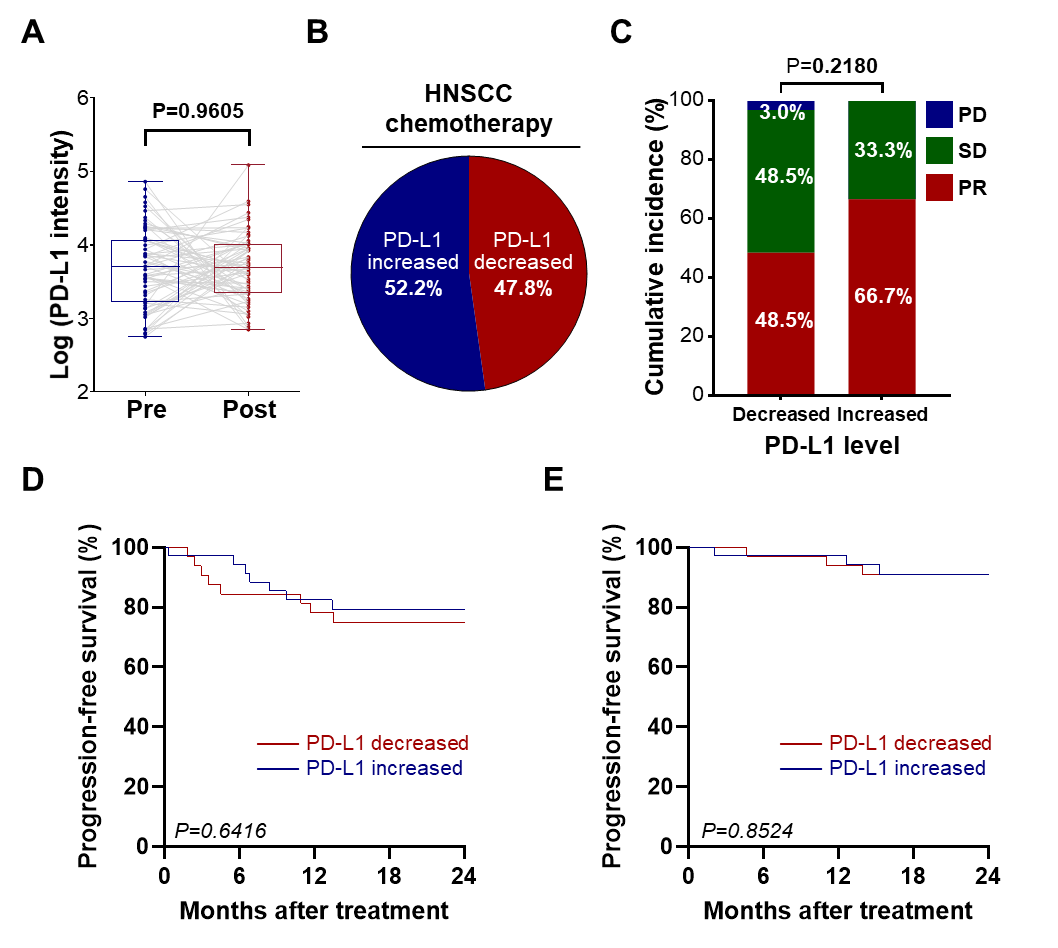


**Figure S23. EpCAM^+^** **EV PD-L1 and outcomes following cytotoxic chemotherapy.** (A) Changes in EpCAM^+^ EV PD-L1 expression in blood during chemotherapy. (B) Distribution of patients with HNSCC with increased (blue) or decreased (red) EpCAM^+^ EV PD-L1 expression after treatment. (C) Best response to chemotherapy according to changes in EpCAM^+^ EV PD-L1 expression in patients with HNSCC. (D) Progression-free survival after treatment with chemotherpy according to changes in EpCAM^+^ EV PD-L1 expression in patients with HNSCC. (E) Overall survival after treatment with chemotherpy according to changes in EpCAM^+^ EV PD-L1 expression in patients with HNSCC.


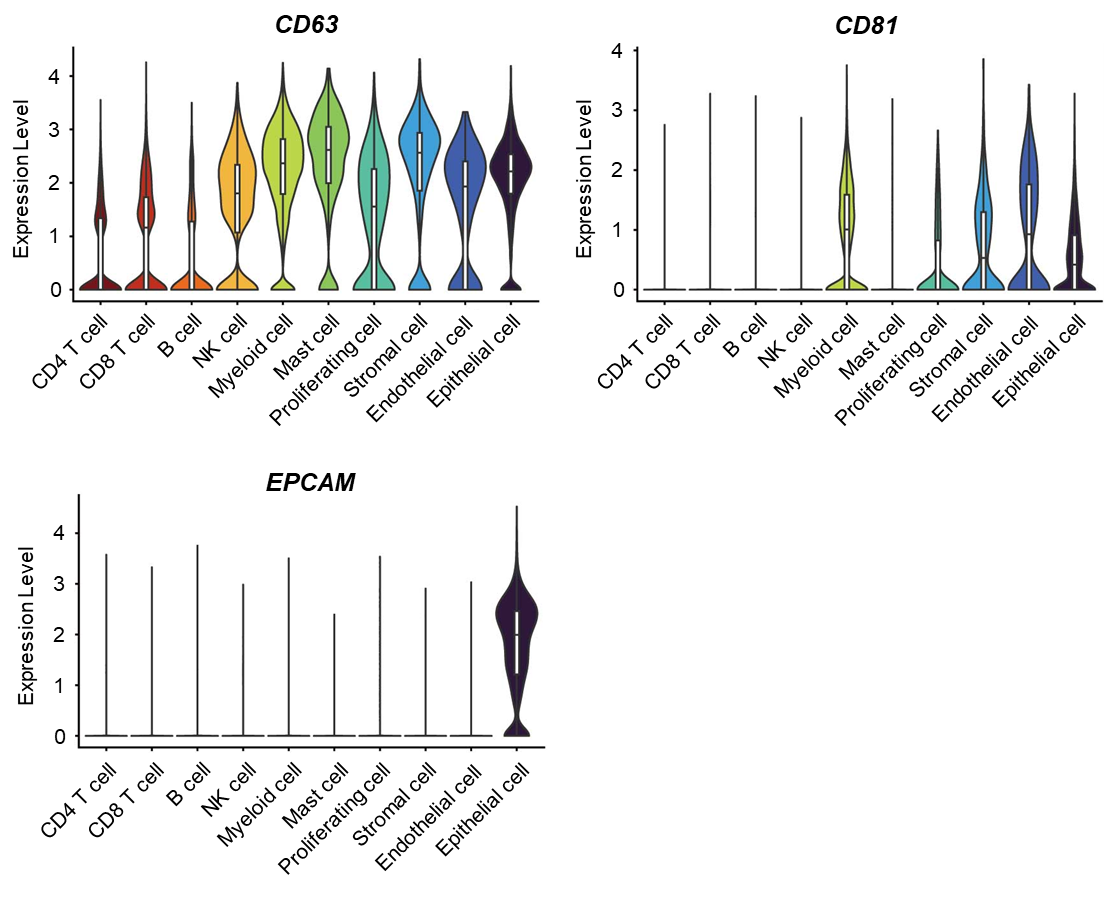


**Figure S24.** Violin plot showing the expression levels of *CD63*, *CD81*, and *EPCAM* across diverse cell subtypes identified in the scRNA-seq data. Each plot is colored by cell subtype.

**Tables**

**Table S1 | Baseline characteristics of the participants.**

| **Characteristic** | **Patients (*n* = 213)** |
| --- | --- |
| Age (years), median (range) | 67 (31–90) |
| Sex, *n* (%) |  |
| Male | 161 (75.6) |
| Female | 52 (24.4) |
| Smoking status, *n* (%) |  |
| Never | 64 (30.1) |
| Former | 88 (41.3) |
| Current | 61 (28.6) |
| Cancer type, *n* (%) |  |
| HNSCC | 73 (34.3) |
| NSCLC | 140 (65.7) |
| Tissue PD-L1 expression (%), *n* (%) |  |
| 0 | 102 (47.9) |
| 1–49 | 74 (34.7) |
| ≥50 | 37 (17.4) |
| Immunotherapy drug, *n* (%) |  |
| Atezolizumab | 77 (36.2) |
| Nivolumab | 121 (56.8) |
| Pembrolizumab | 15 (7.0) |
| Treatment line, *n* (%) |  |
| 1 | 11 (5.1) |
| 2 | 142 (66.7) |
| 3 | 33 (15.5) |
| 4 | 14 (6.6) |
| 5 | 8 (3.7) |
| 6 | 4 (1.9) |
| 7 | 1 (0.5) |

**Table S2.** **Multivariable Cox proportional hazards regression analysis for PFS and OS in patients with HNSCC**

|  |  | **Multivariable analysis for PFS** | |  |  | **Multivariable analysis for OS** | |
| --- | --- | --- | --- | --- | --- | --- | --- |
|  |  | **Hazard ratio (95% CI)** | **P-value** |  |  | **Hazard ratio (95% CI)** | **P-value** |
| Age |  |  | 0.777 |  |  |  | 0.747 |
| <65 years old |  | Reference |  |  |  | Reference |  |
| ≥65 years old |  | 1.081 (0.631-1.853) |  |  |  | 1.098 (0.623-1.934) |  |
| Sex   \| **Sex** \|  \| 0.079 \| \| --- \| --- \| --- \|  \| Female \| Reference \|  \| \| --- \| --- \| --- \|  \| Male \| 1.244 (0.976-1.584) \|  \| \| --- \| --- \| --- \| |  |  | 0.019 |  |  |  | 0.011 |
| Female |  | Reference |  |  |  | Reference |  |
| Male |  | 0.396 (0.183-0.860) |  |  |  | 0.372 (0.173-0.798) |  |
| Smoking |  |  | 0.272 |  |  |  | 0.518 |
| Never smoker |  | Reference |  |  |  | Reference |  |
| Ex or current smoker |  | 1.409 (0.764-2.599)  ) |  |  |  | 1.218 (0.670-2.211) |  |
| Line of treatment |  |  | 0.967 |  |  |  | 0.192 |
| ≤2 |  | Reference |  |  |  | Reference |  |
| ≥3 |  | 1.015 (0.492-2.094) |  |  |  | 1.637 (0.781-3.432) |  |
| Tissue PD-L1 TPS |  |  | 0.183 |  |  |  | 0.278 |
| 0% |  | Reference |  |  |  | Reference |  |
| ≥1% |  | 1.479 (0.832-2.629) |  |  |  | 1.376 (0.773-2.447) |  |
| Exosomal PD-L1 dynamics |  |  | <0.001 |  |  |  | <0.001 |
| Increased |  | Reference |  |  |  | Reference |  |
| Decreased |  | 0.337 (0.189-0.603) |  |  |  | 0.306 (0.169-0.555) |  |

Abbreviations: PFS, progression-free survival; OS, overall survival; CI, confidence interval; TPS, tumor proportion score.

**Table S3. Multivariable Cox proportional hazards regression analysis for PFS and OS in patients with NSCLC**

|  |  | **Multivariable analysis for PFS** | |  |  | **Multivariable analysis for OS** | |
| --- | --- | --- | --- | --- | --- | --- | --- |
|  |  | **Hazard ratio (95% CI)** | **P-value** |  |  | **Hazard ratio (95% CI)** | **P-value** |
| Age |  |  | 0.144 |  |  |  | 0.551 |
| <65 years old |  | Reference |  |  |  | Reference |  |
| ≥65 years old |  | 0.758 (0.522-1.099) |  |  |  | 0.893 (0.614-1.297) |  |
| Sex   \| **Sex** \|  \| 0.079 \| \| --- \| --- \| --- \|  \| Female \| Reference \|  \| \| --- \| --- \| --- \|  \| Male \| 1.244 (0.976-1.584) \|  \| \| --- \| --- \| --- \| |  |  | 0.148 |  |  |  | 0.793 |
| Female |  | Reference |  |  |  | Reference |  |
| Male |  | 0.616 (0.320-1.188) |  |  |  | 0.913 (0.463-1.800) |  |
| Smoking |  |  | 0.705 |  |  |  | 0.842 |
| Never smoker |  | Reference |  |  |  | Reference |  |
| Ex or current smoker |  | 1.132 (0.595-2.156)  ) |  |  |  | 1.072 (0.540-2.126) |  |
| Line of treatment |  |  | 0.029 |  |  |  | 0.101 |
| ≤2 |  | Reference |  |  |  | Reference |  |
| ≥3 |  | 1.517 (1.044-2.202) |  |  |  | 1.385 (0.939-2.043) |  |
| Tissue PD-L1 TPS |  |  | 0.633 |  |  |  | 0.418 |
| 0% |  | Reference |  |  |  | Reference |  |
| ≥1% |  | 0.917 (0.644-1.307) |  |  |  | 1.165 (0.805-1.684) |  |
| Exosomal PD-L1 dynamics |  |  | 0.022 |  |  |  | 0.037 |
| Increased |  | Reference |  |  |  | Reference |  |
| Decreased |  | 0.653 (0.455-0.939) |  |  |  | 0.671 (0.460-0.977) |  |

Abbreviations: PFS, progression-free survival; OS, overall survival; CI, confidence interval; TPS, tumor proportion score.
